# Tau spread plays an etiopathogenic connection between seizures and Alzheimer’s disease

**DOI:** 10.1016/j.isci.2026.117747

**Published:** 2026-11-03

**Authors:** Yanli Jiang, Liulin Wu, Wensheng Li, Longfei Li, Zhizhou Huang, Yuran Gui, Jian-Zhi Wang, Rong Liu, Chao-Hui Jing, Xiaochuan Wang

**Affiliations:** 1 Hubei Key Laboratory of Cognitive and Affective Disorders, School of Medicine, Jianghan University, Wuhan 430056, China; 2 Co-innovation Center of Neuroregeneration, Nantong University, Nantong, Jiangsu 226001, China; 3 Department of Pathophysiology, School of Basic Medicine, Tongji Medical College, Huazhong University of Science and Technology, Wuhan 430030, China; 4 Shenzhen Huazhong University of Science and Technology Research Institute, Shenzhen 518000, China; 5 Department of Neurosurgery, XinHua Hospital, Affiliated to Shanghai JiaoTong University School of Medicine, Shanghai 200092, China

**Keywords:** Alzheimer’s disease, seizures, Tau, spread, Tau pathology

## Abstract

Patients with Alzheimer’s disease (AD) display heightened seizure susceptibility, in which neuronal hyperactivity during seizures correlates with exacerbated Tau pathology and cognitive impairment. This study integrated public transcriptomic datasets with machine learning approaches, suggesting Tau spread as a key biological association between seizure activity and AD. We validated activity-dependent Tau release under epileptic stimulation using primary neurons and human induced pluripotent stem cells (hiPSC)-derived neuronal models. Further validation in an AAV-based mouse Tau propagation model revealed robust bidirectional interactions: pathological Tau overexpression worsened seizure outcomes, while seizures accelerated Tau propagation across brain regions, inducing neuronal loss and gliosis. This study characterizes the activity-regulated interplay between epileptic activity and AD-associated Tau pathology, offering a potential therapeutic target for AD comorbid with epilepsy.

## Highlights

• Integrated transcriptomic reveals shared molecular signatures between seizures and AD
• Neuronal hyperexcitability triggers release and cell-to-cell spread of pathological Tau
• Seizures accelerate Tau spread, neuronal loss and gliosis in hTau-overexpressing mice

## Graphical abstract

**Figure:**
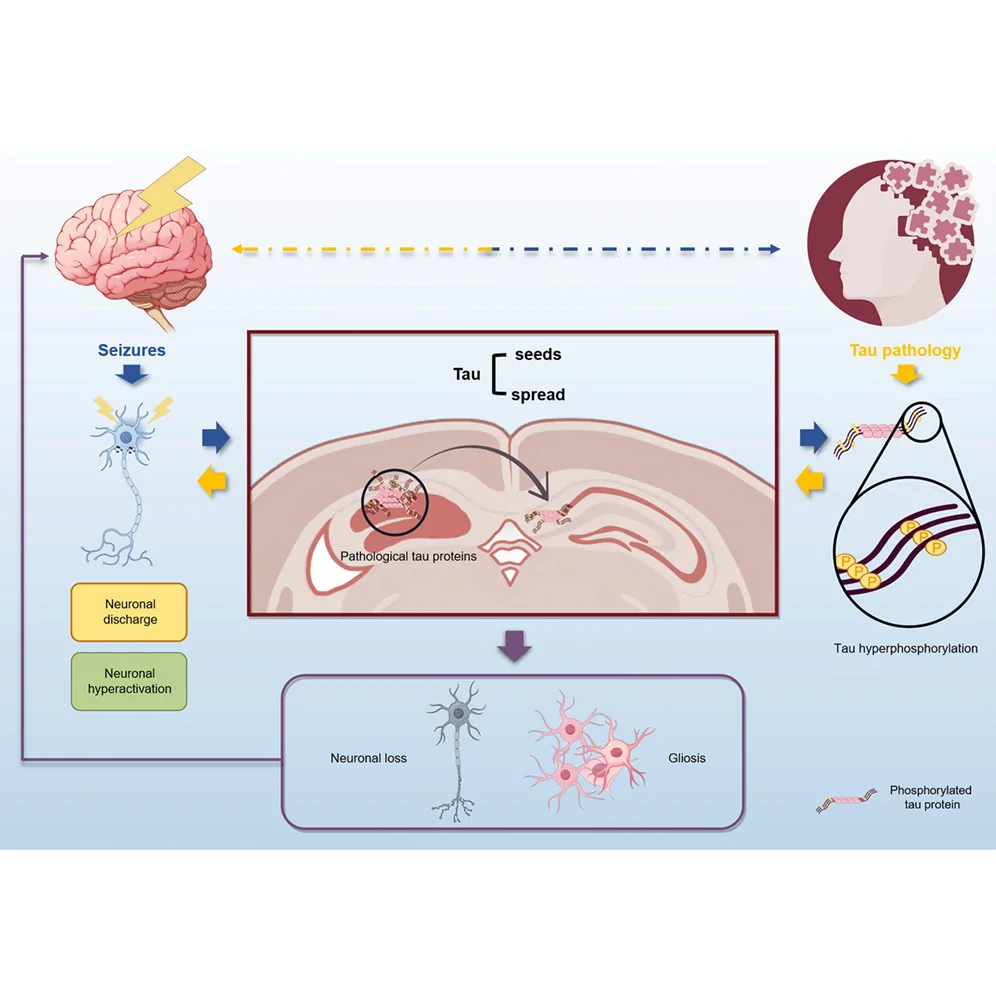

## Teaser

Disease; Biological sciences; Neuroscience

## Introduction

Alzheimer’s Disease (AD), the most common form of dementia, is characterized by intracellular neurofibrillary tangles (NFTs) composed of hyperphosphorylated Tau,^1^^,^^2^ and extracellular amyloid-β (Aβ) plaques.^3^^,^^4^ Neuronal hyperexcitability is observed in the early or even presymptomatic stages of AD,^5^^,^^6^ which could be used to identify individuals at risk of dementia and start treatments before neurodegeneration progresses. People with AD have an increased incidence of seizures even early in the course of the disease compared with the general population, with about 22% of them experiencing seizures.^7^^,^^8^ The simultaneous occurrence of seizures and epileptiform activity exacerbates AD pathologies, including Tau hyperphosphorylation and Aβ deposition.^9^^,^^10^^,^^11^ Poorly controlled seizures represent a risk factor for AD, and antiepileptic drugs have some benefits in AD.^12^^,^^13^ Besides, hyperphosphorylated Tau has been detected in patients with refractory epilepsy.^14^ However, given that AD and seizures share similar pathology and common underlying cellular mechanisms, seizures may be underdiagnosed and are often considered a complication of AD.

Both Aβ and Tau can contribute to neuronal hyperexcitability. For example, Aβ can reduce the expression of voltage-gated potassium channels subfamily 4 (Kv4) and increase N-methyl-D-aspartate receptors (NMDAR) activation via increased D-serine and glutamate release and reduced glutamate uptake.^15^^,^^16^^,^^17^^,^^18^^,^^19^^,^^20^^,^^21^ Missorting and absence of Tau disrupt postsynaptic localization of Fyn, resulting in reduced NMDAR phosphorylation and disrupted NMDAR/PSD-95 interaction. This uncouples NMDAR-mediated excitotoxicity and, hence, mitigates Aβ toxicity.^22^^,^^23^ Moreover, conditional ablation of Tau in excitatory neurons reduces epilepsy in a mouse model of Dravet syndrome.^24^ And targeting hyperphosphorylated Tau by treatment with sodium selenate has anti-seizure effects in a broad range of rodent models.^25^

Tau pathology is initiated in the trans-entorhinal and entorhinal cortex, from where it sequentially progresses to the limbic system including hippocampus and olfactory bulb, and eventually the neocortex in a pattern that suggests spread along neuroanatomical connections.^26^^,^^27^ Studies have shown that pathological Tau aggregates extracted from the AD brain or mutated Tau-expressing mouse brain act as “seeds” to capture normal Tau and template it into filaments,^28^^,^^29^^,^^30^ which is known as a prion-like property. In addition, intracellular Tau can transfer directly between cells through tunneling nanotubes,^31^ and it can be released into the extracellular space, either as free Tau or within exosomes or ectosomes, and taken up by the recipient cells.^32^^,^^33^ Therefore, the transfer of pathological Tau between cells underlies the progression of Tau pathology in the brains of AD patients, ultimately exacerbating cognitive impairments. Many evidences suggest that enhanced neuronal activity stimulates the release and spread of Tau both *in vitro* and *in vivo* and enhances Tau pathology.^34^^,^^35^^,^^36^^,^^37^ In turn, Tau protein regulates neuronal activity,^22^^,^^23^^,^^24^ although the exact mechanism still needs to be explored.

The aim of this study was to investigate whether seizure-like activity promotes pathological Tau release and spread and aggravates Tau pathology. To explore the potential molecular basis of this interaction, we first performed an integrative transcriptomic analysis combining multiple publicly available epilepsy and AD datasets. Differential expression and co-expression network analyses revealed convergent molecular signatures and key pathways shared between seizures and AD, particularly those related to Tau pathology and neurodegeneration. Furthermore, machine learning algorithms consistently prioritized 22 core genes that may underlie this comorbidity and represent potential therapeutic targets. Building upon these bioinformatic findings, we next used the glutamate analog kainic acid (KA) to stimulate neuronal activity *in vitro*, and observed increased release of both endogenous Tau and overexpressed P301L mutant human Tau (hTau (P301L)) into the culture medium from mouse primary neurons and human induced pluripotent stem cells (hiPSC)-derived neurons. We then employed a Tau spread model *in vivo* by stereotactically injecting AAV9-EGFP-P2A-hTau (P301L)-3Flag unilaterally into the hippocampal CA1 region of wild-type (WT) mice. Fifteen days later, KA was administered at the same site to induce medial temporal lobe epilepsy (MTLE). The KA-injected ipsilateral hippocampus serves as the epileptogenic zone with significant sclerosis,^38^ while the contralateral hippocampus represents a seizure propagation zone without sclerosis.^39^ We found that in mice with comorbid Tau pathology and MTLE, hTau (P301L) spread from the injection site to adjacent and contralateral brain regions, where Tau pathology, neuronal loss, and gliosis were detected. Moreover, these comorbid mice had increased seizure severity, mortality, and motor activity compared to mice with MTLE alone. Together, our findings highlight the importance of the pathological Tau spread in AD and MTLE comorbidity.

## Results

### Integrated transcriptomic and machine learning analysis reveals shared molecular signatures between seizures and Alzheimer’s disease

To elucidate the potential molecular links between seizures and AD, we systematically analyzed differentially expressed genes (DEGs) and co-expression networks across relevant transcriptomic datasets. To reduce heterogeneity and enhance analytical power, we first integrated five publicly available transcriptomic datasets (GEO: GSE181804, GSE60862, GSE186334, GSE63808, GSE134697, and GSE84422) encompassing cortical and hippocampal tissue samples from individuals with epilepsy and matched controls. Batch effects were corrected using the ComBat algorithm (Figure 1A). Principal component analysis (PCA) after correction (Figure S1A) confirmed that samples clustered primarily by tissue type and disease condition, indicating successful dataset harmonization.

**Figure 1:**
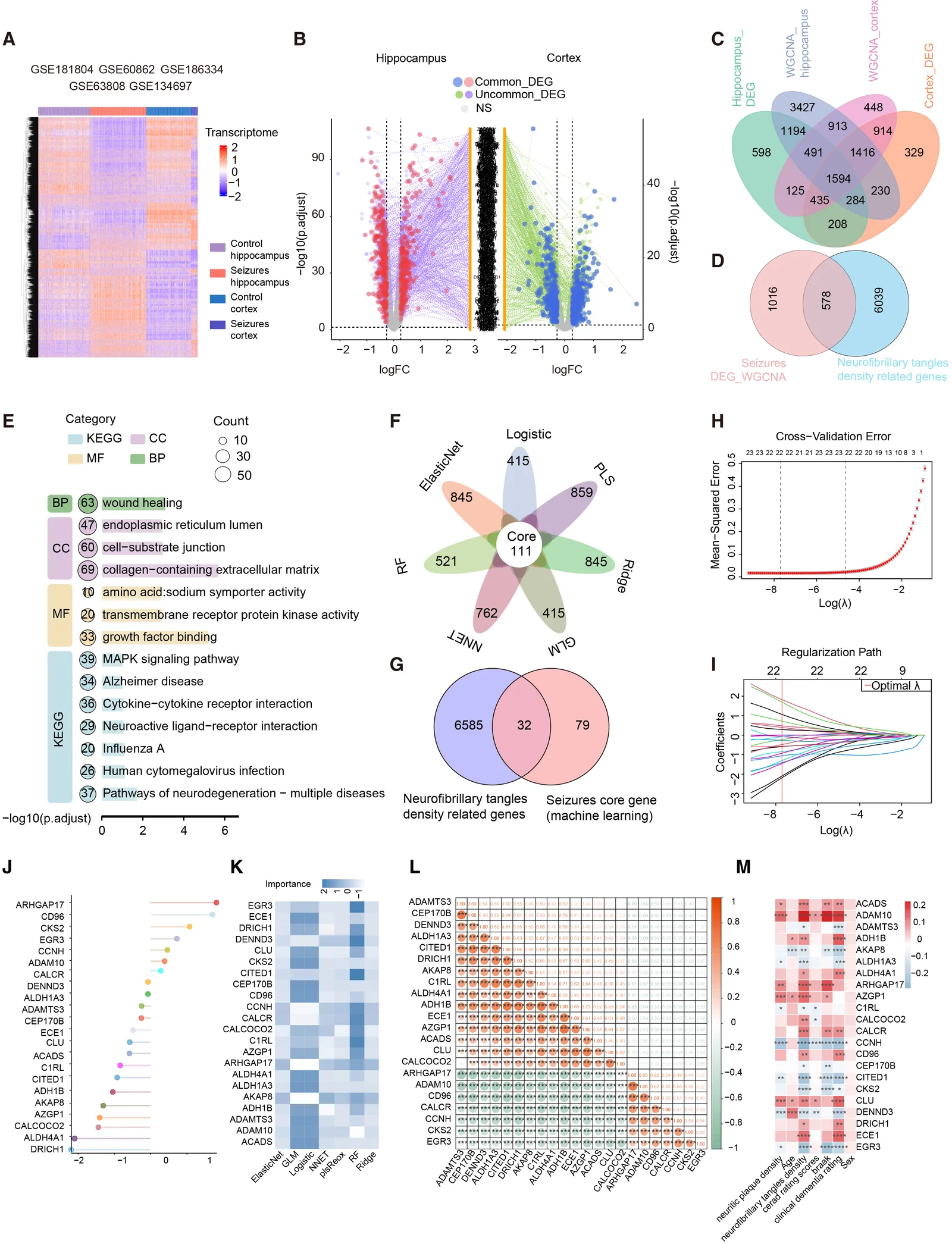
Integration of multiple seizures datasets revealed the relationship between seizures and AD (A) Heatmap of gene expression profiles after integrating five datasets (GEO: GSE181804, GSE60862, GSE186334, GSE63808, and GSE134697), showing distinct transcriptional patterns in hippocampal and cortical tissues under control and seizures. The width of each top annotation bar corresponds to the sample size of each group. (B) Volcano plots of hippocampal and cortical DEGs. DEGs in hippocampus and cortex are each identified by comparing seizure samples against control samples. Purple and green dots indicate region-specific DEGs (Uncommon_DEG) in cortex and hippocampus while pink and blue dots represent shared DEGs between regions (Common_DEG). (C) Venn diagram of four gene sets: hippocampal DEGs (Hippocampus_DEG), hippocampal hub-module genes identified by WGCNA (WGCNA_hippocampus), cortical hub-module genes (WGCNA_cortex), and cortical DEGs (Cortex_DEG). Numbers denote gene counts for each sector. (D) Venn diagram comparing seizure-related DEGs with Tau-pathology-associated genes from GEO: GSE84422; the overlap comprises 578 genes. (E) Gene Ontology: Biological Process (BP), Cellular Component (CC), and Molecular Function (MF) and KEGG enrichment for the intersecting genes (*n* = 1,594). (F) 111 seizure-related core genes selected by 7 machine-learning algorithms: ElasticNet, Logistic regression, Partial Least Squares (PLS), Ridge, random forest (RF), neural network (NNET), and generalized linear model (GLM). (G) Venn diagram comparing the 111 seizure-related core genes with neurofibrillary tangle density related genes from GEO: GSE84422. (H) 5-fold cross-validation curve of the LASSO model. The dashed line marks the λ that minimizes mean-squared error. (I) LASSO regularization path. The gray vertical line corresponds to the optimal λ from (H), leaving 22 genes with non-zero coefficients. (J) Coefficients for the 22 core genes by LASSO algorithms. (K) Heatmap showing the importance of scores of each gene in the 7 machine-learning algorithms, with color and size indicating the level of importance. (L) Correlation heatmap showing pairwise expression correlations among the 22 core genes. (M) Correlation heatmap showing the correlation between core genes and various clinical indicators of AD, with color indicating the strength of correlation in GEO: GSE84422.

We next performed differential gene expression analyses separately for the cortical and hippocampal regions, identifying 2,521 genes consistently differentially expressed in both regions (Figure S1B). Volcano plots illustrated the expression fold change and statistical significance of these DEGs (Figure 1B). To further dissect gene co-regulation patterns, we applied weighted gene co-expression network analysis (WGCNA), which revealed 15 hippocampal and 14 cortical co-expression modules. The top three modules most strongly associated with disease status (control vs. seizure) were designated as WGCNA_hippocampus and WGCNA_cortex modules, respectively (Figures S1C–S1H). Intersecting the hippocampal and cortical DEGs with their corresponding WGCNA hub modules yielded 1,594 high-confidence candidate genes (Figure 1C). To further probe the overlap with AD-specific pathology, we compared this candidate gene set with genes associated with NFT density from the GEO: GSE84422 dataset using Pearson correlation analysis, identifying 578 overlapping genes—representing 36.26% of the candidate set (Figure 1D). Consistent with this, subsequent Kyoto Encyclopedia of Genes and Genomes (KEGG) and Gene Ontology (GO) enrichment analyses indicated that the high-confidence candidate genes were significantly enriched in pathways implicated in AD, neurodegeneration, and neuroactive ligand-receptor interactions (Figure 1E). This substantial intersection suggests shared transcriptional alterations underlying both seizure activity and Tau pathology. Among the 1,594 candidate genes, we next evaluated feature importance using seven different machine learning algorithms, identifying 111 core genes through intersection of the results (Figure 1F). Of these, 32 genes were found to be closely related to Tau pathology (Figure 1G). To further refine this set, we performed least absolute shrinkage and selection operator (LASSO) regression with cross-validation, ultimately identifying 22 core genes (Figures 1H–1J). These 22 genes exhibited high importance scores across all seven algorithms (Figure 1K) and showed strong inter-gene expression correlations (Figure 1L). Validation using the external dataset GEO: GSE84422 demonstrated that most of the core genes were significantly positively correlated with Braak stage or NFT density (Figure 1M), suggesting that these genes may represent molecular convergent points linking neuronal hyperexcitability with Tau dysregulation. Based on these findings, we speculated that Tau spread may underlie the pathological overlap between seizures and AD, and further validated this hypothesis in subsequent experiments.

### Kainic acid-induced increase in neuronal activity promotes the release of normal Tau and pathological Tau

To determine whether KA-induced neuronal activity promotes the release of endogenous Tau, we treat mouse primary neurons with different concentrations of KA (0, 75, 150, and 200 μM) for 24 h. Neuronal activity was assessed by cell immunofluorescence staining with c-Fos antibody. We found that all concentration of KA induced elevated c-Fos expression, which is consistent with increased neuronal activity (Figures 2A and 2B), without altering neuronal viability (Figures 2M and S2). Next, mouse primary neurons and hiPSC-derived neurons (Figure S3) were treated with KA (0, 75, 150, and 200 μM) for 24 h, intracellular Tau was detected by western blotting and Tau secreted into the culture medium was measured by ELISA (Figure 2C). We found that KA stimulation significantly reduced intracellular human Tau (hTau) (Figures 2D and 2E) and mouse Tau (mTau) (Figures 2H–2J), but only 150 μM and 200 μM kA significantly increased the amount of hTau (Figure 2F) and mTau released into the medium compared to the control group (Figure 2K). Moreover, Tau protein levels released into the conditioned medium were negatively correlated with intracellular Tau protein levels (Figure 2G). To rule out the confounding effect of KA-induced cytotoxicity, we performed western blot analysis of cleaved caspase-3 (Figures 2H and 2L) and quantitative immunofluorescence analysis of NeuN-positive neurons in mouse primary neurons (Figure S2). Lactate dehydrogenase (LDH) cytotoxicity assays were also conducted in both mouse primary neurons and hiPSC-derived neurons (Figures 2M and 2N). Compared with control groups, no upregulation of cleaved caspase-3 and no obvious loss of NeuN-positive neurons were observed following treatment with 150 μM KA. Consistently, LDH assays confirmed that the tested concentrations of KA exerted no harmful effects on the viability of mouse primary neurons. Collectively, these findings demonstrate that elevated Tau levels in the culture medium arise from active cellular secretion, rather than passive leakage from damaged or dying.

**Figure 2:**
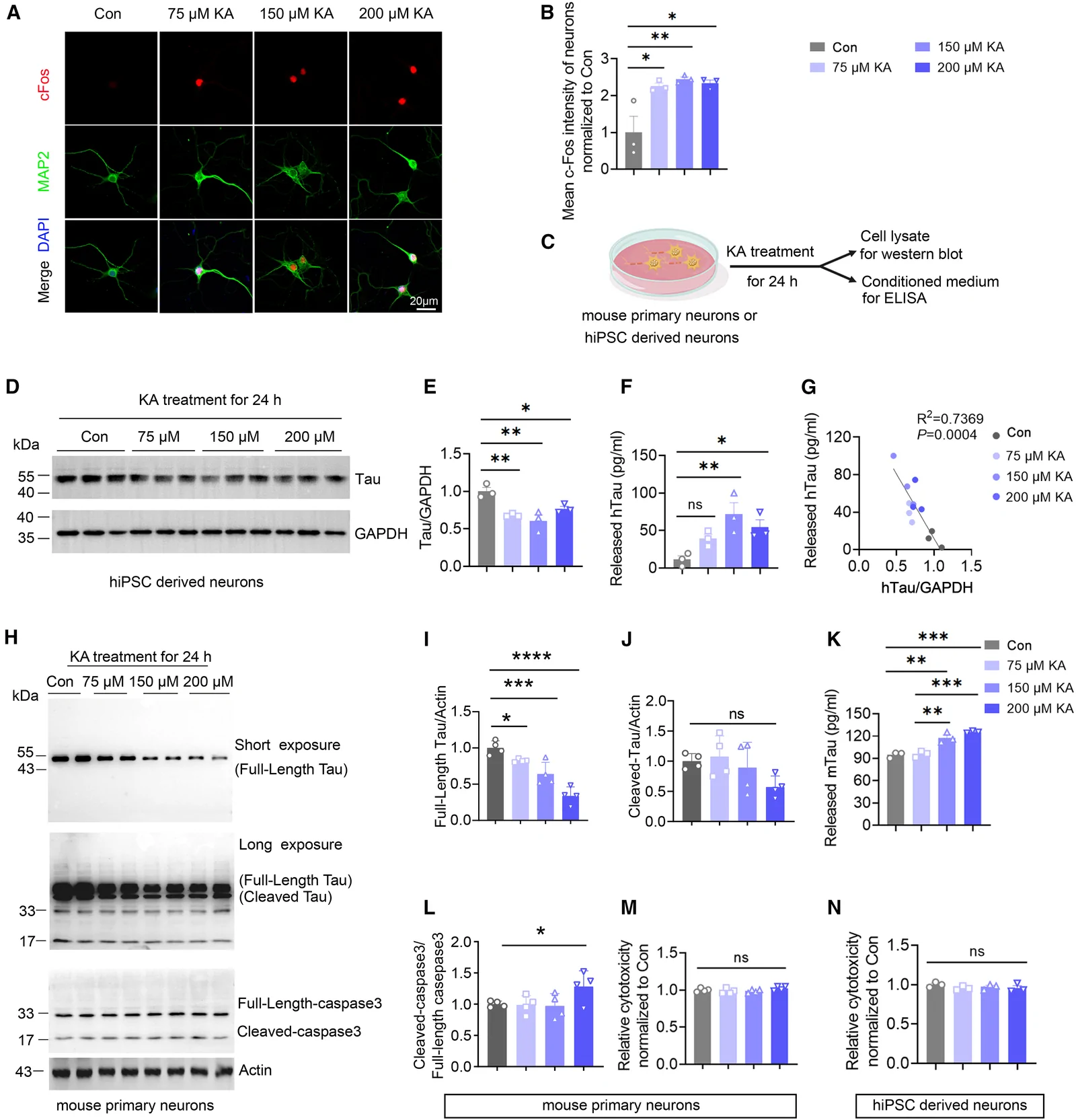
KA-induced increase in neuronal activity of mouse primary neurons and hiPSC-derived neurons promotes endogenous Tau release into the culture medium (A) Mouse primary neurons were treated with different concentrations of KA (0, 75, 150, and 200 μM) for 24 h, then immunofluorescence staining was performed with antibodies against c-Fos (red) and MAP2 (green). Scale bars, 20 μm. (B) Mean fluorescence intensity of c-Fos was quantified and normalized to the control. (C) Experimental procedure diagram: KA was used to treat mouse primary neurons and hiPSC-derived neurons, and then the cell lysates and conditioned medium were collected for western blot and ELISA, respectively. (D) hiPSC-derived neurons were treated with different concentrations of KA for 24 h, then western blotting was performed with antibodies against Tau and GAPDH. (E) Endogenous human Tau (hTau) protein level in hiPSC-derived neurons was quantified and normalized to the control. (F) ELISA quantification of secreted hTau in the conditioned medium from hiPSC-derived neurons following 24 h kA treatment. (G) Linear regression analysis revealing a negative linear correlation between intracellular hTau level (normalized to GAPDH) and extracellular released hTau content in KA-exposed hiPSC-derived neurons. (H) Western blot results from KA gradient-treated mouse primary neurons (24 h): short exposure for full-length Tau, long exposure for full-length and cleaved truncated Tau fragments, as well as full-length-caspase-3 and cleaved caspase-3. (I and J) Quantitative statistical analysis of full-length Tau/actin and (J) cleaved-Tau/actin ratios across different KA concentration groups. (K) ELISA quantification of secreted murine Tau (mTau) in the conditioned medium from mouse primary neurons following 24 h kA treatment. (L) Quantitative analysis of the cleaved-caspase-3/full-length caspase-3 ratio in each group. (M) Cytotoxicity of mouse primary neurons treated with different concentrations of KA for 24 h was assessed by LDH assay. (N) Cytotoxicity of hiPSC-derived neurons treated with different concentrations of KA for 24 h was assessed by LDH assay. Significant differences were determined using one-way ANOVA followed by Tukey’s multiple comparison test (*n* = 3–4 per group). Data are presented as the mean ± SEM, with ∗*p* < 0.05, ∗∗*p* < 0.01, ∗∗∗*p* < 0.001, and ∗∗∗∗*p* < 0.0001, ns, no significance.

Additionally, we observed decreased absolute phosphorylation levels of Tau at the S199, S214, and S396 sites when normalized to GAPDH following KA treatment. Gradient KA stimulation experiments further demonstrated a dose-dependent elevation in the pS199-Tau/total Tau ratio. Further quantification of the pTau/total Tau ratio at three phosphorylation sites revealed a significant increase after KA exposure, indicating that the relative proportion of phosphorylated Tau in the intracellular Tau pool was robustly raised despite the global reduction in total intracellular Tau protein (Figure S4). To verify whether this reduction in intracellular phosphorylated Tau was due to secretion into the extracellular space instead of intracellular dephosphorylation or degradation, we measured phosphorylated Tau in the medium by ELISA (Figure S5). The results verified that KA stimulation significantly elevated phosphorylated Tau levels in the culture medium, directly proving that the drop in intracellular phosphorylated Tau at these key phosphorylation sites is caused by augmented secretion of phosphorylated Tau into the culture medium.

To explore whether neuronal activity affects pathological Tau release, we infected mouse primary neurons with AAV9 (Adeno-associated virus serotype 9)-CMV (Cytomegalovirus)-EGFP-P2A-hTau (P301L)-3Flag, which is widely used to study Tau spread.^40^^,^^41^^,^^42^ The P2A peptide self-cleaves during translation,^43^ so the virus can express two proteins, EGFP and hTau (P301L)-3Flag (Figures 3A and 3B). A small amount of EGFP-P2A-hTau (P301L)-3Flag protein was also detected due to incomplete self-cleavage of P2A (Figure 3B). We used AAV9-CMV-EGFP-P2A-3Flag as a control (3Flag group). Six days after virus infection, mouse primary neurons were treated with 150 μM kA for 24 h. We found that KA treatment had no effect on intracellular EGFP and EGFP-P2A-3Flag protein levels in 3Flag or hTau (P301L) group (Figures 3B–3D), but induced a significant decrease in intracellular hTau (P301L) protein level (Figures 3B–3E and 3F) and an increase in the amount of hTau released into the medium in hTau (P301L) group (Figure 3G). These results further rule out the possibility that KA induces Tau release by causing cell death.

**Figure 3:**
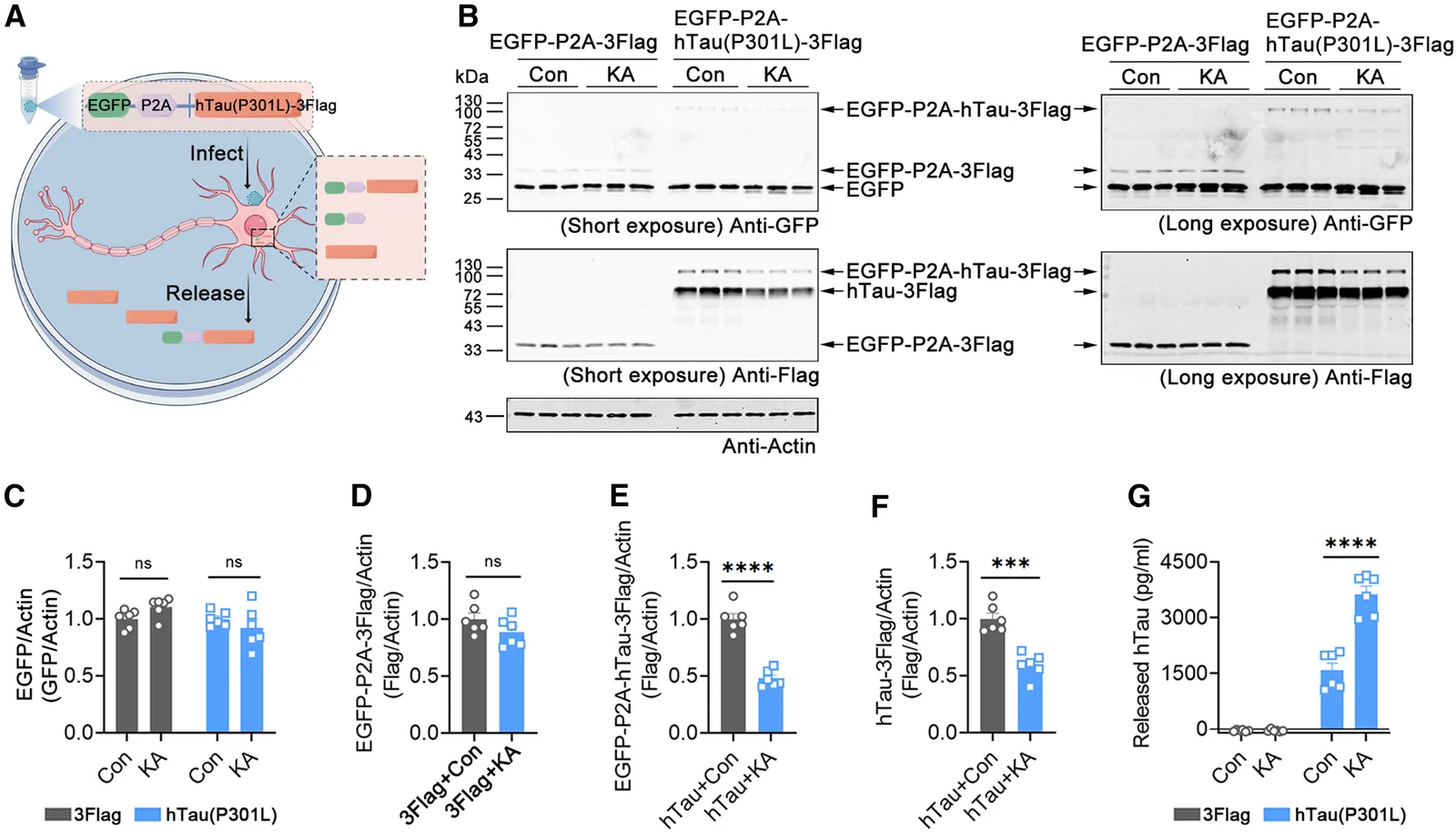
KA promotes the release of pathological hTau (P301L) from mouse primary neurons into the culture medium (A) Schematic: mouse primary neurons were infected with AAV9-CMV-EGFP-P2A-hTau (P301L)-3Flag, producing three proteins (EGFP-P2A-hTau (P301L)-3Flag, EGFP-P2A, and hTau (P301L)-3Flag). hTau (P301L)-containing proteins were released into the conditioned medium. (B) Mouse primary neurons were infected with AAV9-CMV-EGFP-P2A-3Flag or AAV9-CMV-EGFP-P2A-hTau (P301L)-3Flag for 6 days, then were treated with or without KA (0 μM or 150 μM) for 24 h, and intracellular protein levels of EGFP-P2A-hTau (P301L)-3Flag, hTau (P301L)-3Flag, EGFP-P2A, EGFP-P2A-3Flag, and GAPDH were detected by western blotting with antibodies against GFP, Flag, and GAPDH. (C and D) Intracellular protein levels of EGFP-P2A and EGFP-P2A-3Flag in (B) were quantified. (E and F) Intracellular protein levels of EGFP-P2A-hTau (P301L)-3Flag and hTau (P301L)-3Flag in (B) were quantified. (G) hTau protein levels (EGFP-P2A-hTau (P301L)-3Flag and hTau (P301L)-3Flag) released into the conditioned medium were measured by ELISA. Significant differences were determined using unpaired *t* test (*n* = 6 per group). Data are presented as the mean ± SEM, with ∗∗∗*p* < 0.001, and ∗∗∗∗*p* < 0.0001, ns, no significance.

Altogether, these findings indicate that KA-induced increase in neuronal activity promotes endogenous Tau and pathological Tau release into extracellular medium.

### Pathological Tau increases KA-induced seizures severity and mortality in mice

To investigate the interplay between Tau pathology and epilepsy *in vivo*, we administered AAV9-CMV-EGFP-P2A-hTau (P301L)-3Flag (hTau (P301L) group) or AAV9-CMV-EGFP-P2A-3Flag (3Flag group) into the unilateral hippocampal CA1 region in WT mice. Fifteen days later, KA (250 ng) was injected at the same site to induce MTLE (Figure 4A). Local field potentials (LFPs) traces from the ipsilateral hippocampi of mice with KA injection showed epileptiform discharges (Figure 4B). According to the modified Racine score, seizure severity was significantly increased in the hTau (P301L) group compared to the 3Flag group within two days after KA injection (Figure 4C), but there was no difference in seizure latency between the two groups (Figure 4D). Furthermore, seizures resulted in a significant decrease in the survival rate of mice in the hTau (P301L) group. While the seizure-induced mortality in the hTau (P301L) group was higher than in the 3Flag group, no statistically significant difference was observed (Figure 4E). These results demonstrate that pathological Tau increases KA-induced seizure severity and mortality in mice.

**Figure 4:**
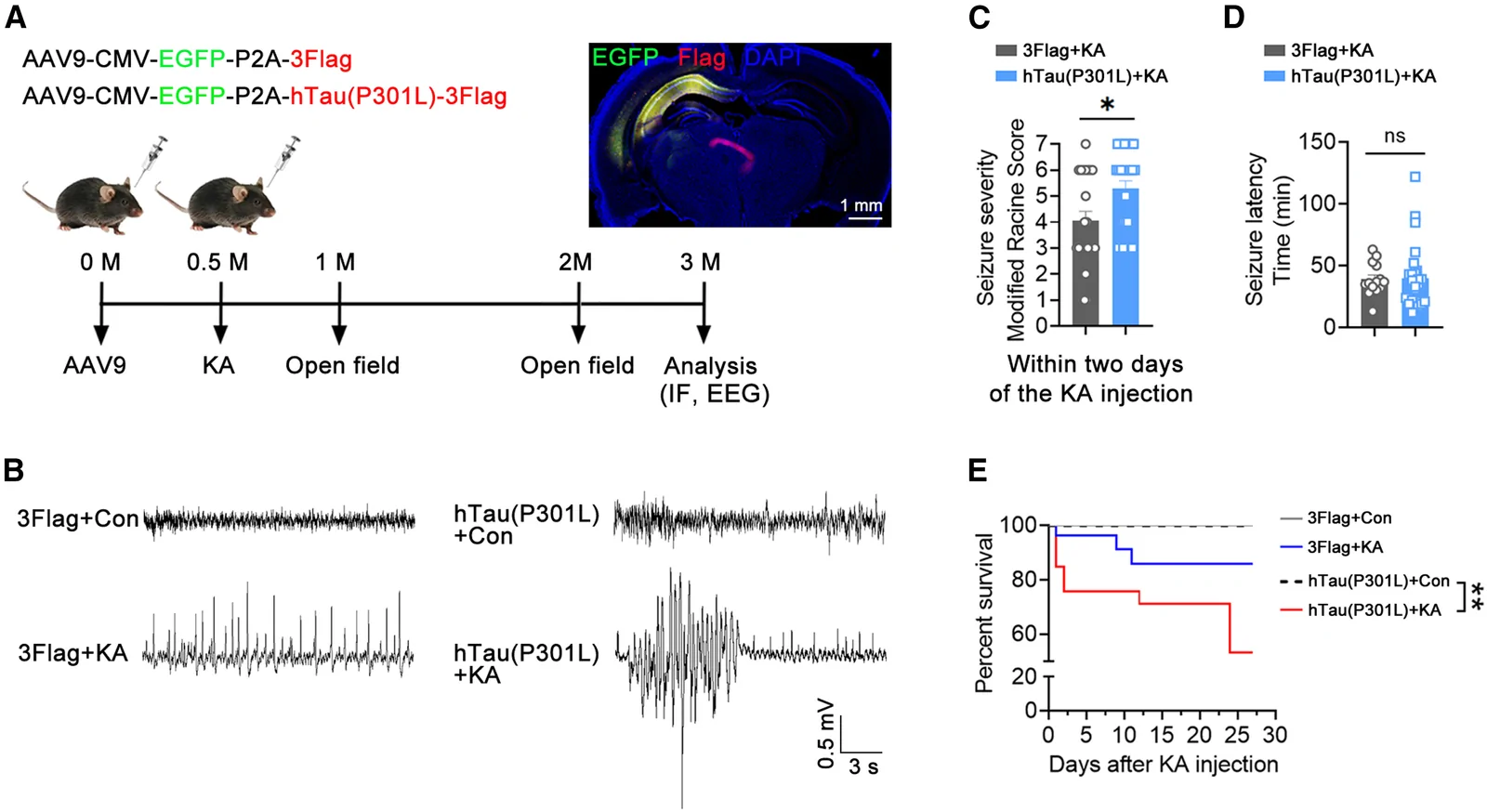
KA-induced seizure severity and mortality are significantly increased in hTau (P301L)-overexpressing mice (A) Schematic of the experimental procedure. AAV9-CMV-EGFP-P2A-3Flag or AAV9-CMV-EGFP-P2A-hTau (P301L)-3Flag were unilaterally injected into the right hippocampal CA1 region in 8-week-old C57BL/6 male mice. Fifteen days after virus injection, KA was injected into the same site. Behavioral tests were performed 1 month and 2 months after virus injection. After 3 months of virus injection, immunofluorescence staining was performed and LFPs in the hippocampus were recorded. (B) Raw representative LFP traces from mice expressing 3Flag or hTau (P301L) with or without KA injection. (C) Maximum Racine score of mice in 3Flag group and hTau (P301L) group within 2 days after KA injection. (D) Latency of seizures in mice expressing 3Flag or hTau (P301L) after KA injection (3Flag + KA, *n* = 23; hTau (P301L) + KA, *n* = 25). (E) Kaplan-Meier survival curve of mice expressing 3Flag or hTau (P301L) with or without KA injection (3Flag + Con, *n* = 18; 3Flag + KA, *n* = 23; hTau (P301L) + Con, *n* = 18; hTau (P301L) + KA, *n* = 28). Significant differences were determined using unpaired *t* test or two-way ANOVA followed by Sidak’s multiple comparison test. Survival differences were calculated by the Log rank (Mantel-Cox) test. Data are presented as the mean ± SEM, with ∗*p* < 0.05, ∗∗*p* < 0.01, ns, no significance.

In addition, one month after virus injection, KA-induced seizures markedly reduced the time spent in the central square by mice in the hTau (P301L) group but not in the 3Flag group (Figures S6A and S6B), indicating increased anxiety. The overall distance traveled and speed in the open field test remained unchanged among the four groups, indicating unaffected general motor ability (Figures S6C and S6D). However, two months after hTau (P301L) expression, mice with MTLE spent more time in the central square than mice without MTLE (Figure S6E), possibly due to increased motor activity reflected in the increase in distance traveled and speed (Figures S6F and S6G).

### Seizures promote hTau (P301L) spread to adjacent and contralateral brain regions, inducing phosphorylated pathological Tau in the contralateral hippocampus

To explore whether seizure-like activity promotes pathological Tau spread, we stained brain slices with Flag antibody to show the expression of hTau (P301L). According to the AAV-based Tau spread model (AAV9-CMV-EGFP-P2A-hTau (P301L)-3Flag), transduced neurons express EGFP and hTau (P301L)-3Flag (donor cells, GFP^+^) and can be discriminated from neurons only expressing hTau (P301L)-3Flag due to Tau spread (recipient cells, Flag^+^/GFP^−^). The number of Flag^+^/GFP^−^ cells can reflect the spread of hTau (P301L).^41^^,^^42^ There was no Flag^+^/GFP^−^ cells in either the proximal or distal brain regions of 3Flag-expressing mice with or without MTLE (Figures 5A–5C), which ruled out the effects of viral diffusion and cell death. In hTau (P301L)-expressing mice, only a small amount Flag^+^/GFP^−^ cells were found in adjacent and contralateral brain regions, but seizures induced a significant increase in the number of Flag^+^/GFP^−^ cells in ipsilateral and contralateral brain regions (Figures 5A–5C). Altogether, in hTau (P301L)-expressing mice, hTau (P301L) can spread to neurons in distal anatomically connected regions, and seizures exacerbate its spread.

**Figure 5:**
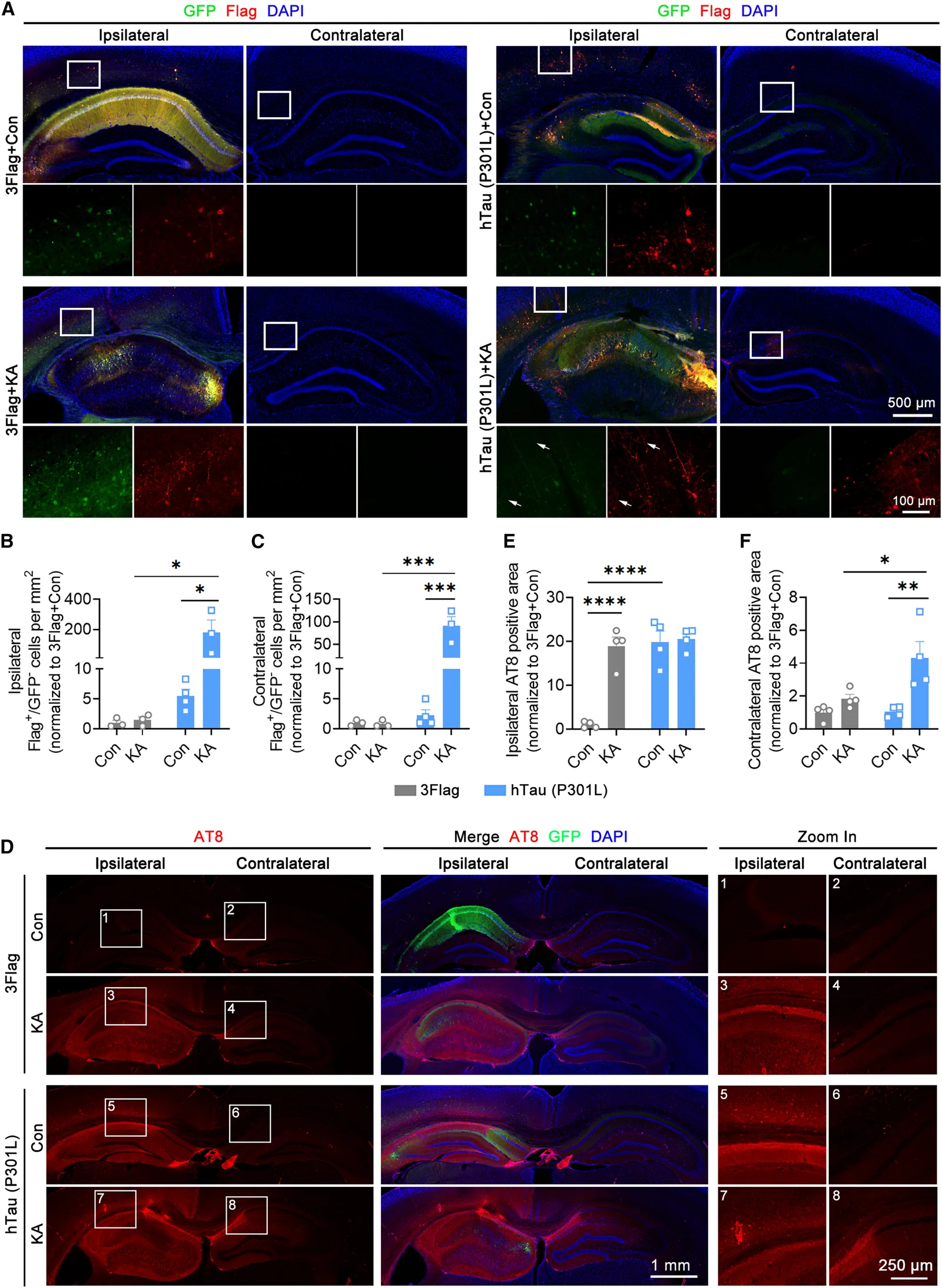
Seizures promote hTau (P301L) spread to adjacent and contralateral brain regions and induce Tau pathology in the contralateral hippocampus in hTau (P301L)-overexpressing mice (A) Brain slices of mice overexpressing EGFP-P2A-3Flag or EGFP-P2A-hTau (P301L)-3Flag with or without KA injection were immune-stained with Flag antibody. GFP-positive cells (GFP^+^) are hTau (P301L) donor cells in which the virus is expressed, while Flag-positive and GFP-negative cells (Flag^+^/GFP^−^) are recipient cells for hTau (P301L)-3Flag transmission. Arrows indicate Flag^+^/GFP^−^ cells. Scale bars, 500 μm and zoom in images, bar = 100 μm. (B) Quantification of Flag^+^/GFP^−^ cells per mm^2^ in the ipsilateral hippocampus and cortex. (C) Quantification of Flag^+^/GFP^−^ cells per mm^2^ in the contralateral hippocampus and cortex. (D) Brain slices of mice expressing EGFP-P2A-3Flag or EGFP-P2A-hTau (P301L)-3Flag with or without KA injection were immune-stained with AT8. Scale bars, 1 mm and zoom in images, bar = 250 μm. (E) AT8-positive areas in the ipsilateral hippocampus were quantified and normalized to 3Flag + Con group. (F) AT8-positive areas in the contralateral hippocampus were quantified and normalized to 3Flag + Con group. Significant differences were determined using two-way ANOVA followed by Sidak’s multiple comparison test (*n* = 3–4 mice per group; 3–4 brain slices per mouse). Data are presented as the mean ± SEM, with ∗*p* < 0.05, ∗∗*p* < 0.01, ∗∗∗*p* < 0.001, and ∗∗∗∗*p* < 0.0001.

Then, we stained mouse brain slices with AT8 antibody to detect the phosphorylation of Tau in the hippocampus (Figure 5D). AT8 antibody recognizes paired helical filaments-Tau (PHF-Tau, mTau and hTau) phosphorylated at Ser202 and Thr205. AT8 positive areas were significantly increased in the ipsilateral hippocampus of mice with MTLE in both 3Flag group and hTau (P301L) group compared to WT control group (Figures 5D and 5E). Although in the hTau (P301L) group, there was no significant difference in AT8 positive area in the ipsilateral hippocampus between mice with MTLE and mice without MTLE (Figures 5D and 5E), seizures promoted a significant increase in AT8 positive area in the contralateral hippocampus of mice expressing hTau (P301L) but not 3Flag (Figures 5D and 5F). Together, these results suggest that seizures induce phosphorylated pathological Tau in the contralateral hippocampus of hTau (P301L)-expressing mice, which may be caused by seizures-induced spread of hTau (P301L) to the contralateral hippocampus.

### Neuronal loss and gliosis are aggravated in mice with Tau pathology and MTLE comorbidity

To investigate neurodegeneration, we stained mouse brain sections with the neuronal marker NeuN to identify neuronal loss (Figure 6A). NeuN-positive areas and mean intensity in the ipsilateral hippocampus of mice with MTLE in both 3Flag group and hTau (P301L) group were significantly decreased compared to those in WT group (Figures 6A, 6B, and S7A). In the hTau (P301L) group, seizures did not exacerbate neuronal loss in the ipsilateral hippocampus (Figures 6A, 6B, and S7A), but induced a significant decrease in the NeuN-positive area in the contralateral hippocampus, without affecting the mean intensity of NeuN (Figures 6A, 6C, and S7B). Additionally, we stained mouse brain sections with the microglial marker Iba1 and the astrocyte marker GFAP to detect neuroinflammation (Figure 6D). The Ibal- and GFAP-positive areas, as well as the mean intensity in the ipsilateral hippocampus, were significantly increased in both the 3Flag group with MTLE and the hTau (P301L) group with or without MTLE (Figures 6D–6F, S7C, and S7D). In the hTau (P301L) group, seizures induced a further increase in the GFAP-positive area and the mean intensity of Iba1 in the ipsilateral hippocampus (Figures 6D–6F and S7C). Moreover, seizures induced a significant elevation in Iba1-and GFAP-positive areas, as well as in the mean intensity of GFAP, in the contralateral hippocampus of mice expressing hTau (P301L), but not in those expressing 3Flag (Figures 6D–6G, 6H, S7E, and S7F). Microglial activation was further confirmed by the morphological analysis, characterized by larger cell bodies, fewer branches, and shorter process length (Figure S8). Together, these results indicate that neuronal loss and gliosis are aggravated in mice with Tau pathology and MTLE comorbidity.

**Figure 6:**
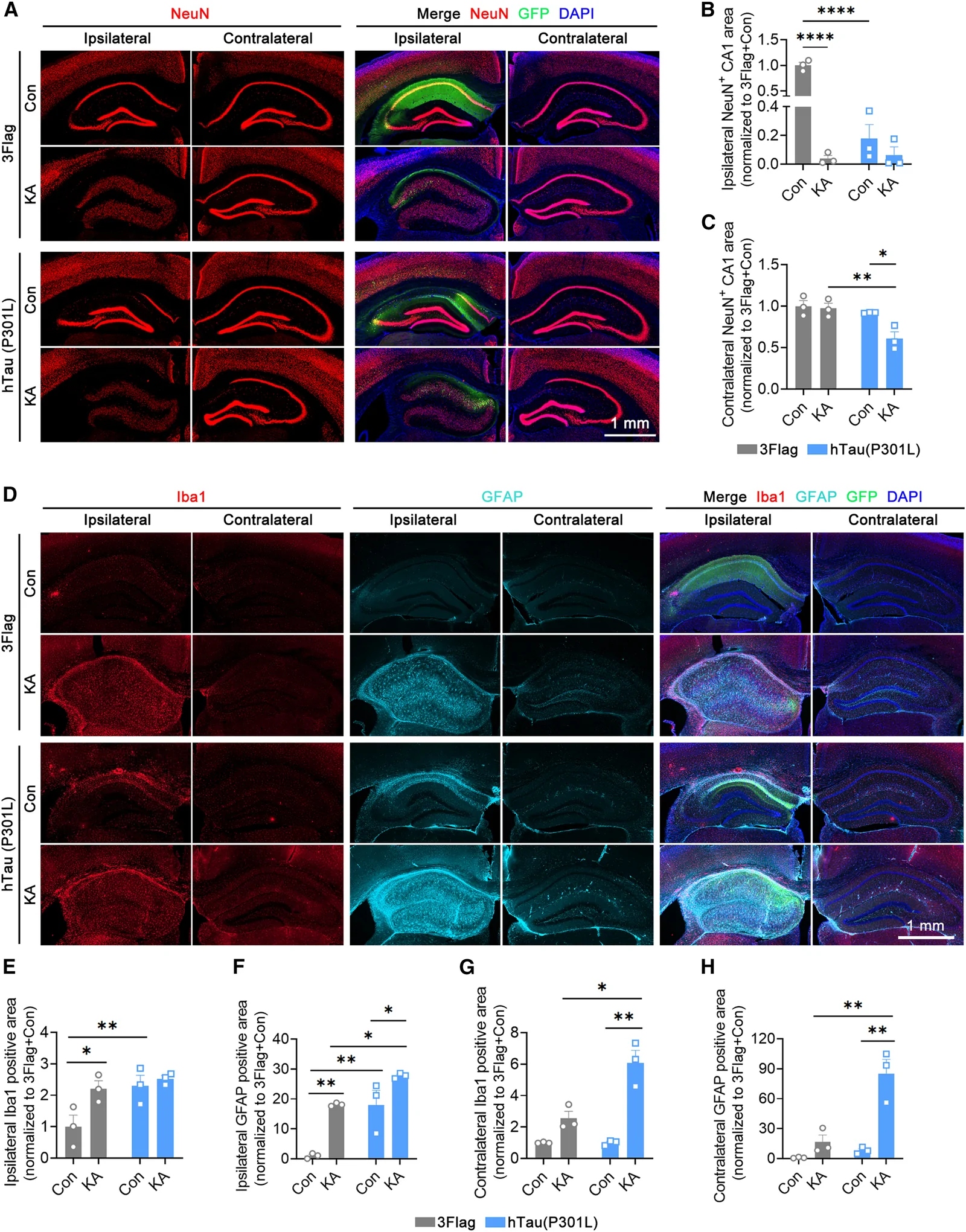
Seizures induce neuronal loss and gliosis in the contralateral hippocampus of hTau (P301L)-overexpressing mice (A) Brain slices of mice expressing 3Flag or hTau (P301L) with or without KA injection were immune-stained with NeuN. Scale bars, 1 mm. (B and C) NeuN-positive areas in the ipsilateral and contralateral hippocampal CA1 regions were quantified and normalized to 3Flag + Con group. (D) Brain slices of mice expressing 3Flag or hTau (P301L) with or without KA injection were immune-stained with Ibal and GFAP. Scale bars, 1 mm. (E and F) Iba1-and GFAP-positive areas in the ipsilateral hippocampal CA1 regions were quantified and normalized to 3Flag + Con group. (G and H) Iba1-and GFAP-positive areas in the contralateral hippocampal CA1 regions were quantified and normalized to 3Flag + Con group. Significant differences were determined using two-way ANOVA followed by Sidak’s multiple comparison test (*n* = 3 mice per group; 3–4 brain slices per mouse). Data are presented as the mean ± SEM, with ∗*p* < 0.05, ∗∗*p* < 0.01, and ∗∗∗∗*p* < 0.0001, ns, no significance.

Collectively, these data suggest that seizure activity accelerates pathological Tau spread, which in turn exacerbates neurodegenerative changes and gliosis, indicating a potential link between seizure activity and AD-related Tau pathology (Figure 7).

**Figure 7:**
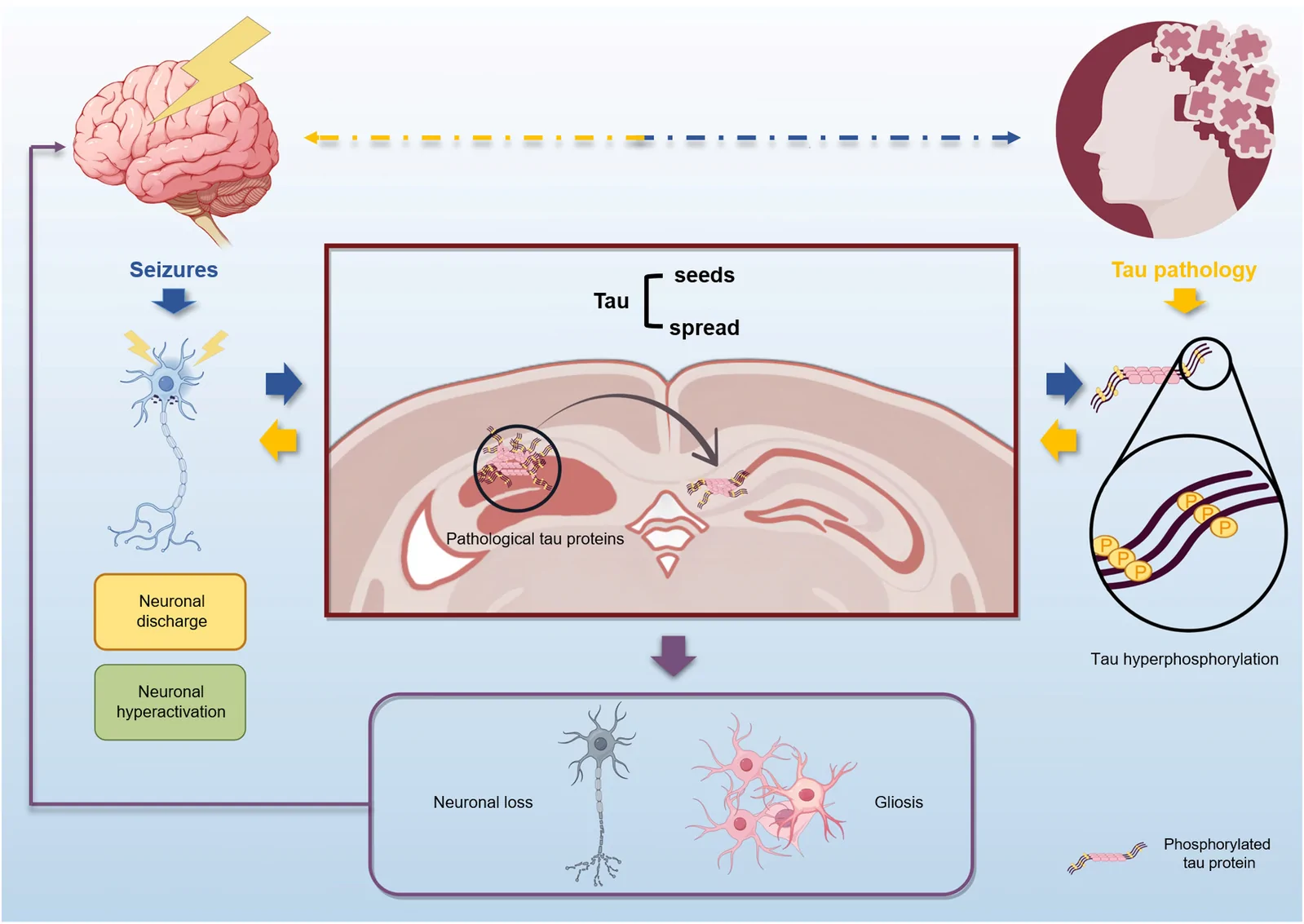
A proposed novel etiopathogenic mechanism of seizures and AD Tau pathology comorbidity, which is initiated by Tau spread Schematic illustration shows that seizures-induced abnormal neuronal discharges and hyperactivation lead to the release and spread of pathological Tau protein, thereby promoting the phenotype of AD Tau pathologies, including abnormal hyperphosphorylation of Tau, neuronal loss, and gliosis. Subsequently, on the one hand, Tau hyperphosphorylation accelerates the abnormal neuronal discharges and the release and spread of Tau protein induced by KA, and on the other hand, neuronal loss and gliosis exacerbate the phenotype of seizures, forming a vicious cycle that ultimately leads to the comorbidity of seizures and AD Tau pathologies. The diagram was drawn on the FigDraw scientific drawing platform.

## Discussion

In this study, we sought to explore the connection between epilepsy and AD. First, we provide converging evidence from integrative transcriptomic analyses. By integrating multiple publicly available epilepsy and AD datasets, we identified convergent molecular signatures and co-expression modules that link seizure-related neuronal hyperactivity with neurodegenerative processes, particularly those involving Tau pathology. Machine learning algorithms further prioritized 22 core genes that may play central roles in this interaction. Among these 22 genes, several candidates have established links with neuronal activity, Tau metabolism, neuroinflammation, and cellular stress responses. For example, EGR3 is an activity-dependent immediate-early transcription factor induced during epileptic activity and involved in neuronal excitability-related transcriptional responses.^44^^,^^45^ ADAM10 functions as the major neuronal α-secretase and regulates synaptic protein processing.^46^ CLU is an Alzheimer’s disease risk gene involved in extracellular protein homeostasis and neuroinflammatory regulation.^47^ Furthermore, CALCOCO2/NDP52 participates in selective autophagy and has been reported to facilitate phosphorylated Tau clearance.^48^^,^^49^ ALDH1A3 is associated with glial activation^50^ and γ-aminobutyric acid (GABA) metabolism.^51^ C1RL and CD96 are involved in immune-related signaling pathways^52^^,^^53^ and may contribute to neuroinflammatory responses. These findings provide a biological basis linking our transcriptomic signature with KA-induced neuronal hyperactivity, Tau release, neuronal loss, and gliosis.

Further validation of these candidate genes in independent clinical cohorts will be important in future studies. Then, using mouse primary neurons and hiPSCs-derived neurons, we demonstrated that endogenous mTau or hTau and pathological P301L-hTau can be released into the extracellular medium, which is consistent with the known fact that intracellular Tau, including both normal Tau and pathological Tau, can be secreted into the extracellular space and taken up by the recipient cells.^32^^,^^33^ This, coupled with the fact that pathological Tau can work as seeds to recruit and induce endogenous Tau aggregation and phosphorylation in a prion-like manner,^29^^,^^30^ are the basis for the progression of Tau pathology along anatomically connected brain regions in AD patients. It has been reported that elevated neuronal excitability promotes the release and spread of Tau,^34^^,^^35^^,^^36^^,^^37^ which supports our finding that neurons with higher c-Fos immunoreactivity tend to exhibit increased Tau release. In this study, since the effects of KA in the conditional medium cannot be ruled out, we did not explore whether the KA-induced increase in the amount of hTau secreted into the conditional medium promoted the uptake of Tau by recipient cells. However, considering that extracellular Tau is capable of cell-to-cell transmission via the culture medium,^35^ it is reasonable to speculate that increased Tau secretion into the medium may facilitate greater Tau internalization by recipient cells.

The phosphorylation of Tau at Ser199, Ser214, and Ser396 sites decreased in mouse primary neurons after KA treatment, possibly due to the reduction of total Tau protein. Following KA treatment in mouse primary neurons, only the released pathological Tau, not the endogenous normal Tau, acts as seeds to recruit normal Tau and template it into filaments, thereby exacerbating Tau pathology. Additionally, in cultured primary neurons expressing hTau (P301L), all neurons become hyperexcitable and release hTau (P301L) into the culture medium upon KA treatment. However, it is only when this released hTau (P301L) is taken up by normal neurons that it induces phosphorylation of endogenous Tau. Thus, the decrease in Tau phosphorylation in cultured primary neurons treated with KA is a result of the sustained release of total Tau. This limitation underscores the challenges of using this method to study the spread of pathological Tau.

*In vivo* animal model, we overexpressed hTau (P301L) in the unilateral hippocampal CA1 region to induce Tau pathology, and 15 days later injected KA at the same site to model MTLE. Using this mouse model, we analyzed the interplay between Tau pathology and epilepsy. We found that hTau (P301L)-expressing mice with MTLE had a further increase in seizure severity, including increased mortality belonging to Racine stage 7. This is consistent with the positive correlation between increased phosphorylated Tau in the hippocampus and increased seizure frequency in MTLE patients.^54^ Although AD patients are much more susceptible to epilepsy than normal population,^7^^,^^8^ and Tau pathology increases the incidence of seizures,^25^ there was no difference in KA-induced seizure latency between hTau (P301L)-expressing mice and 3Flag-expressing mice in our study, which may be related to the length of time hTau (P301L) is expressed before KA injection.

Epilepsy is often comorbid with anxiety, and many anticonvulsants or antiepileptic drugs also have anxiolytic effects, depending largely on the dose used.^55^^,^^56^ In our study, we also observed increased anxiety in the KA + hTau (P301L) group of mice, without any changes in locomotor ability, one month after virus injection. But two months after hTau (P301L) overexpression, mice with MTLE spent more time in the central square than mice without MTLE, which may be caused by increased travel distance and speed. Several studies have reported that epileptic mice exhibit abnormally high locomotor activity and elevated exploratory behavior in the open field test.^57^^,^^58^^,^^59^ Heterozygous Scn1a^WT/R613X^ mice with spontaneous seizures traveled longer distances and at higher velocities compared to their WT littermates in the open field test.^60^ This increased hyperactivity of epileptic mice may be due to the inhibitory deficits in neural circuits responsible for controlling their locomotor activity and exploratory behavior. The exact mechanism of increased locomotor activity in epileptic mice needs further study.

Enhanced neuronal activity induced optogenetically or chemogenetically exacerbates Tau pathology by promoting the release and spread of Tau.^34^^,^^35^ Epilepsy is a neurological disorder characterized by recurrent seizures caused by abnormal neuronal discharges or hyperexcitability of neurons with synchronicity.^61^ Seizures promote Tau hyperphosphorylation,^10^ and seizures frequency is associated with enhanced phosphorylated Tau pathology in AD patients.^62^ In this study, we found that seizures promote the spread of hTau (P301L) to adjacent and contralateral brain regions, which is responsible for phosphorylated pathological Tau, neuronal loss, and gliosis in the contralateral hippocampus in hTau (P301L)-overexpressing mice with MTLE.

KA treatment leads to an increase in intracellular calcium level.^63^ Importantly, Tau can be cleaved by Ca^2+^-activated calpain^64^ and can also be hyperphosphorylated by cyclin-dependent kinase 5 (Cdk5), which is activated by associating with its regulatory subunit, p35. The binding of p25 (a calpain-cleaved form of p35) to Cdk5 constitutively activates Cdk5, further promoting Tau hyperphosphorylation.^65^ Therefore, the potential dysregulation of calcium levels induced by KA treatment may also contribute to accelerated pathological Tau spreading. Further studies investigating calpain activity and the relative levels of p25 and p35 following KA treatment would be worthwhile.

### Limitations of the study

This study has several recognizable limitations that should be acknowledged. First, although neuronal models confirmed activity-dependent Tau release, the residual pharmacological effect of KA in conditioned medium could not be completely excluded, leaving the causal link between secreted Tau and subsequent cellular uptake unclarified. Second, the observed reduction in Tau phosphorylation in cultured neurons reflects sustained Tau extracellular secretion rather than decreased pathological modification, suggesting limitations of neuronal culture systems for modeling long-term Tau propagation pathology. Third, the evaluation of Tau pathology relied solely on AT8 staining, which cannot differentiate soluble phosphorylated Tau from insoluble fibrillary aggregates. Without complementary gold-standard staining methods for mature NFTs, the full formation of seed-competent Tau aggregates in remote brain regions cannot be conclusively verified. Fourth, only mutant P301L Tau was used in the current propagation model; the lack of WT human Tau control prevents clarification of whether seizure activity specifically accelerates pathological Tau spread over physiological Tau. Finally, the calcium-dependent calpain-Cdk5 signaling pathway was only indirectly inferred, and further quantitative validation is required to confirm its regulatory role in activity-driven Tau propagation.

## Resource availability

### Lead contact

Requests for further information and resources should be directed to and will be fulfilled by the lead contact, Xiaochuan Wang (wangxiaochuan@jhun.edu.cn).

### Materials availability

All unique reagents generated in this study (AAV9-hTau(P301L) virus) are available from the lead contact upon reasonable request with a completed materials transfer agreement. No new plasmids or mouse lines were deposited to public repositories.

### Data and code availability

• The transcriptomic datasets used for identifying seizure- and Tau-associated genes were downloaded from the GEO: GSE181804, GSE60862, GSE186334, GSE63808, and GSE134697. Original experimental data have been deposited in Mendeley data: https://doi.org/10.17632/9vj3rdwdtm.1.
• The R scripts (including the machine learning models and the pipeline for normalizing the several GEO datasets) supporting the findings of this study are openly available in GitHub: https://github.com/LWS521/SZ and archived at Zenodo data: https://doi.org/10.5281/zenodo.22722779.
• Any additional information required to reanalyze the data reported in this work paper is available from the lead contact upon request.

## Acknowledgments

We thank the Medical Subcenter of HUST Analytical & Testing Center for data acquisition. The authors are grateful to Mr. Dan Ke and Ms. Qun Wang for helpful technical suggestions during the conduct of this study. This work is supported in parts by grants from 10.13039/501100001809National Natural Science Foundation of China (U25A2074 and 82330041), 10.13039/501100004791Shenzhen Medical Research Fund (B2402037), 10.13039/501100010877Science, Technology and Innovation Commission of Shenzhen Municipality (JCYJ20240813153427036).

## Author contributions

X.W. designed, planned, and organized all experiments and results, including the writing of the manuscript. Y.J., L.W., and W.L. planned and performed all the experiments. L.L., Z.H., and Y.G. analyzed and interpreted the data. J.-Z.W. and R.L. proofread the manuscript. C.-H.J. organized results and interpreted the data. All authors read and approved the final manuscript.

## Declaration of interests

The authors have no relevant financial or non-financial interests to disclose.

## STAR★Methods

### Key resources table

**Table:** 

REAGENT or RESOURCE	SOURCE	IDENTIFIER
**Antibodies**		

Tau5 (Mouse)	Millipore	Cat# MAB361; RRID: AB_94944
Tau (Rabbit)	ZENBIO	Cat# R25862; RRID: N/A
FLAG Tag (Mouse)	ZENBIO	Cat# 390002; RRID: N/A
GAPDH (Mouse)	ABclonal	Cat# AC033; RRID: AB_2769570
GFP/eGFP Tag (Rabbit)	ZENBIO	Cat# 300943; RRID: N/A
pS199-Tau (Rabbit)	ZENBIO	Cat# R30276; RRID: N/A
pS214-Tau (Rabbit)	ZENBIO	Cat# R30278; RRID: N/A
pS396-Tau (Rabbit)	ZENBIO	Cat# R381213; RRID: N/A
Goat anti-mouse IgG - HRP	ZSGB-BIO	Cat# PV-6002; RRID: AB_2864334
Goat anti-rabbit IgG - HRP	ZSGB-BIO	Cat# PV-6001; RRID: AB_2864333
c-Fos (Mouse)	Santa cruz	Cat# Sc-166940; RRID: AB_10609634
MAP2 (Rabbit)	ABclonal	Cat# A17409; RRID: AB_2770260
DYKDDDDK tag (Rabbit)	Proteintech	Cat# 80010-1-RR; RRID: AB_2882940
AT8 (Mouse)	Invitrogen	Cat# MN1020; RRID: AB_223647
NeuN (Rabbit)	Cell signaling	Cat# 24307; RRID: AB_2651140
Iba1/AIF-1 (Rabbit)	Cell signaling	Cat# 17198; RRID: AB_2820254
GFAP (Mouse)	Cell signaling	Cat# 3670; RRID: AB_561049
Goat anti-mouse IgG-Cy3	Jackson Immuno Research	Cat# 115–165-003; RRID: AB_2338680
Goat anti-rabbit IgG-Cy3	Jackson Immuno Research	Cat# 111–165-003; RRID: AB_2338000
Goat anti-mouse IgG-Cy5	Jackson Immuno Research	Cat# 115–175-146; RRID:AB_2338713
Goat anti-rabbit IgG-Cy5	Jackson Immuno Research	Cat# 111–175-144; RRID: AB_2338013

**Bacterial and virus strains**		

AAV9-CMV-EGFP-P2A-MAPT(P301L)-3Flag	GeneChem	N/A
AAV9-CMV-EGFP-P2A-3Flag	GeneChem	N/A

**Chemicals, peptides, and recombinant proteins**		

Poly-D-lysine	Sigma-Aldrich	Cat# P6407
B-27 supplement	Gibco	Cat# 17504044
Penicillin/Streptomycin	Gibco	Cat# 15140163
Glutamax	Gibco	Cat# 35050061
Kainic Acid	MedChemExpress	Cat# HY-N2309
Vitronectin	Gibco	Cat# A14700
Essential 8^TM^ Medium	Gibco	Cat# A1517001
0.25% Trypsin-EDTA	Gibco	Cat# 25200114
Dispase	STEMCELL	Cat# 07913
*N*-2 supplement	Gibco	Cat# 17502048
Nonessential Amino Acids	Gibco	Cat# 11140050
β-mercaptoethanol	Sigma-Aldrich,	Cat# 60–24-2
Recombinant Human Basic Fibroblast Growth Factor	MedChemExpress	Cat# HY-P7004
DMH1	TOCRIS	Cat# 4126
SB431542	TOCRIS	Cat# 1614
Matrigel	CORNING	Cat# 356234
Accutase	Gibco	Cat# A1110501
4% Paraformaldehyde	Biosharp	Cat# BL539A

**Critical commercial assays**		

LDH Cytotoxicity Assay Kit	Beyotime	Cat# C0016
BCA Protein Assay Kit	Beyotime	Cat# P0012
ECL western blot substrate	Thermo Scientific	Cat# 32209
Human Tau ELISA Kit	Invitrogen	Cat# KHB0041
Mouse Tau ELISA Kit	Elabscience	Cat# E-EL-M1121
Mouse Tau (Phospho) [pS199] ELISA Kit	Invitrogen	Cat# KMB7041

**Deposited data**		

Human seizure hippocampus & cortex RNA-seq	NCBI Gene Expression Omnibus	GEO: GSE181804; GEO: GSE60862; GEO: GSE186334; GEO: GSE63808; GEO: GSE134697
Deposited data	Zenodo data	Zenodo data: https://doi.org/10.5281/zenodo.22722779
Original experimental data	Mendeley data	Mendeley data: https://doi.org/10.17632/9vj3rdwdtm.1
Human Alzheimer’s disease temporal cortex RNA-seq	NCBI Gene Expression Omnibus	GSE84422

**Experimental models: Cell lines**		

Human Induced Pluripotent Stem Cells (hiPSCs)	Kindly provided by Professor Jianzhi Wang’s laboratory	N/A

**Experimental models: Organisms/strains**		

C57BL/6J mice	Hubei Biont Biological Technology Co., Ltd.	MGI:2159769

**Software and algorithms**		

ImageJ	ImageJ	https://imagej.nih.gov/ij/
Neuroexplorer v3.0	Nex Technologies	https://www.neuroexplorer.com/
GraphPad Prism 8	GraphPad	https://www.graphpad.com/scientific-software/prism/

**Other**		

DMEM/F12	Gibco	Cat# 11320033
Neurobasal medium	Gibco	Cat# 21103049
Polyvinylidene difluoride membrane	Millipore	Cat# IPVH00010
Automatic slice-scanning system	Olympus	SV200
Confocal laser scanning microscope	Zeiss	Carl LSM 800
OmniPlex Neural Data Acquisition System	Plexon	N/A

### Experimental model and study participant details

#### Animal models

Male C57BL/6J mice (8–9 weeks old, 20–27 g) were purchased from Hubei Biont Biological Technology Co., Ltd. (Wuhan, China). Mice were housed in animal room with 12-h light/dark cycles and provided with adequate food and water. The mice were gently touched at least once a day for a week before the behavioral experiments. All animal experiments were conducted in accordance with the protocol approved by the Institutional Animal Care and Use Committee at Tongji medical school of Huazhong university of science and technology (IACUC Number: 4649).

#### Mouse primary neuron culture

Mouse primary cortical and hippocampal neurons were prepared from day 16–18 embryos from pregnant C57BL/6J mice. Cortex and hippocampus were collected and digested for 15 min in 0.25% trypsin/EDTA with gently shaking every 5 min at 37°C. The digestion was terminated with DMEM/F12 (Gibco, 11320033) medium containing 10% fetal bovine serum (FBS). Cell suspensions were filtered through a 70 μm filter, and then plated with DMEM/F12 containing 10% FBS on culture dishes precoated with poly-D-lysine (Sigma-Aldrich, P6407). After 5 h, the plating medium was changed to Neurobasal medium (Gibco, 21103049) containing 2% B-27 supplement (Gibco, 17504044), 1×penicillin/streptomycin (Gibco, 15140163), and 1×Glutamax (Gibco, 35050061). Half of the medium was changed every 3–4 days.

Neurons were treated with AAV9 or kainic acid (KA, MedChemExpress, HY-P7331) on day 6 *in vitro* (DIV). A total of 2 μL of AAV9-CMV-EGFP-P2A-3Flag or AAV9-CMV-EGFP-P2A-hTau (P301L)-3Flag (titer: 1.24E+13 vg/mL, OBiO Tech, Inc.) was added to each well of a 6-well plate. After six days, neurons were treated with 150 μM kA for 24 h. Cell lysates and culture medium were then collected for western blotting and ELISA, respectively.

#### Culture and differentiation of human induced pluripotent stem cells (hiPSCs)-derived neurons

Differentiation of human induced pluripotent stem cells (hiPSCs) into neural progenitor cells (NPCs), and neural differentiation of NPCs were performed as described previously.^66^ Briefly, hiPSCs were maintained on vitronectin (Gibco, A14700)-coated culture plate with complete Essential 8^TM^ Medium (Gibco, A1517001). The medium was changed daily, and the cells were passaged every 5–6 days using 0.25% Trypsin-EDTA (Gibco, 25200114). NPCs were generated from hiPSCs by dual inhibition of the SMAD signaling pathway using DMH1 and SB431542. After three times passages, hiPSCs were digested with dispase (STEMCELL, 07913) and cultured in suspension in neural progenitor cells induction medium (NIM) (1:1 vol/vol mixture of DMEM/F12 and neurobasal medium, 1×*N*-2 supplement (Gibco, 17502048), 1×B-27 supplement, 1×nonessential amino acids (NEAA, Gibco, 11140050), 1×GlutaMax, 1×penicillin/streptomycin, 100 μM β-mercaptoethanol (Sigma-Aldrich, 60–24-2), 5 ng/mL recombinant human basic fibroblast growth factor (rhbFGF, MedChemExpress, HY-P7331)), which was supplemented with 2 μM DMH1 (TOCRIS, 4126) and 2 μM SB431542 (TOCRIS, 1614). The medium was changed daily. On day 10, the formed embryonic bodies (EBs) were plated on vitronectin-coated plates in NIM supplemented with 2 μM DMH1 and 2 μM SB431542. The medium was changed daily. Neural rosettes were formed after 6 days, then picked neural rosettes manually and expanded in suspension in neural maintenance medium (NMM) (1:1 vol/vol mixture of DMEM/F12 and neurobasal medium, 1×*N*-2 supplement, 1×B-27 supplement, 1×NEAA, 1×GlutaMax, 1×penicillin/streptomycin, 10 ng/mL rhbFGF, 10 ng/mL rhEGF), forming neurospheres.

To obtain human neurons, neurospheres were plated on the Matrigel (CORNING, 356234)-coated dishes and cultured in neural differentiation medium (NDM) (1:1 vol/vol mixture of DMEM/F12 and neurobasal medium, 1×N2 supplement, 1×B27 supplement, 1×NEAA, 1×GlutaMax, 1×penicillin/streptomycin) supplemented with 0.2 mM ascorbic acid (MedChemExpress, HY-14649) and 25 μM β-mercaptoethanol. The medium was changed daily. The cells were passaged every 6–7 days using accutase (Gibco, A1110501) and cultured for about 7 weeks.

hiPSC-derived mature neurons were identified through immunofluorescence staining with an anti-MAP2 antibody before KA treatment (Figure S3).

No established cell lines were used in this study. We used primary mouse neurons and human induced pluripotent stem cell (iPSC)-derived neurons. All cell preparations were routinely tested for mycoplasma contamination, and no mycoplasma contamination was detected in the cells used in this study.

### Method details

#### Viral vectors

Recombinant adeno-associated virus serotype 9 (AAV9) vectors were custom-synthesized by GeneChem Co., Ltd. (Shanghai, China). The experimental vector, designated AAV9-CMV-EGFP-P2A-MAPT(P301L)-3Flag consistently throughout the manuscript, was constructed on a pAAV backbone driven by the CMV promoter, carrying an EGFP-P2A-MAPT(P301L)-3Flag expression cassette followed by a WPRE element.

A P2A ribosomal skip peptide was inserted between EGFP and P301L mutant human tau to mediate stoichiometric co-expression of the two proteins. The expression profile and cleavage efficiency of the vector were validated by Western blot. The control vector, designated AAV9-CMV-EGFP-P2A-3Flag in line with the naming convention of this study, shared the identical pAAV backbone and expression element configuration, and was used as the empty control.

#### Lactate dehydrogenase (LDH) assay

To assess cell viability, the release of LDH was measured using the LDH Cytotoxicity Assay Kit (Beyotime, C0016) following the manufacturer’s instructions. Each measurement was carried out in triplicate and each experiment was repeated independently for reproducibility.

#### Western blotting

Cells were lysed with RIPA lysis buffer (50 mM Tris (pH 7.4), 150 mM NaCl, 1% Triton X-100, 1% sodium deoxycholate, 0.1% SDS, and 1×cocktail) on ice. Protein concentration was measured by the BCA method (Beyotime, P0012). Samples were subjected to SDS-PAGE and transferred onto a polyvinylidene difluoride membrane (PVDF, Millipore, IPVH00010). The membranes were blocked with TBS (Tris-buffered saline) containing 5% fat-free milk for 30 min at room temperature, then incubated with primary antibodies at 4°C overnight. Membranes were washed with TBST (TBS with 0.1% Tween 20) three times, and incubated with horseradish peroxidase (HRP)-conjugated secondary antibody for 2 h at room temperature. The membranes were finally washed with TBST, incubated with the ECL western blot substrate (Thermo Scientific, 32209), and then exposed to ChemiScope (Clinx Science instruments Co. Ltd). Immunostaining was quantified using Multi Gauge software V3.0 from Fuji Film (Minato, Tokyo, Japan).

#### ELISA analysis

Cell culture medium was collected and centrifuged at 2000 g for 20 min at 4°C to precipitate dead cells and insoluble cell debris. The supernatant was collected, and human Tau protein levels and mouse Tau protein levels were measured using Human Tau ELISA Kit (Invitrogen, KHB0041), mouse Tau ELISA Kit (Elabscience, E-EL-M1121) and Mouse Tau (Phospho) [pS199] ELISA Kit(Invitrogen, KMB7041) according to the manufacturer’s instruction, respectively.

#### Hippocampal virus injection

Anesthetized C57BL/6J male mice were placed in a stereotaxic frame and injected with AAV9-CMV-EGFP-P2A-3Flag or AAV9-CMV-EGFP-P2A-hTau (P301L)-3Flag (titer: 1.24E + 13 vg/mL, OBiO Tech, Inc.). Right hippocampal CA1 region (posterior: −2.5 mm, lateral: 2.0 mm, and ventral: −1.8 mm relative to the bregma) received 2 μL virus at the rate of 0.25 μL/min. The microinjector was left in place for 5 min before slowly withdrawn to prevent leakage. After the skin was sutured, each mouse was placed on a heating blanket until it began to recover, and finally were returned to the home cages.

#### Kainic acid-induced mesial temporal lobe epilepsy model in mice

Fifteen days after the virus injection, 1 μL PBS or KA (250 ng/μL, MCE, HY-P7331) was injected into the same site as the virus (posterior: −2.5 mm, lateral: 2.0 mm, and ventral: −1.8 mm relative to the bregma) at a flux rate of 0.25 μL/min for a total period of 4 min. The microinjector was left in place for 5 min before slowly withdrawn to prevent leakage. The postoperative mice were closely monitored within 2 days, and their behavior was assessed by the modified Racine scale.^67^ Meanwhile, the latency of seizures was recorded. Epileptic mice in the 3Flag + KA group.

Scores: −1. Normal Baseline; 0. Whisker trembling; 1. Sudden behavioral arrest; 2. Facial jerking; 3. Neck jerks; 4. Clonic seizure (sitting); 5. Clonic, tonic-clonic seizure (lying on belly); 6. Clonic, tonic-clonic seizure (lying on side) & wild jumping; 7. Tonic extension, possibly leading to respiratory arrest and death.

#### Electrode implantation and local field potential (LFP) recording

Four-channel electrode was implanted into ipsilateral or contralateral hippocampus (posterior: −2.5 mm, lateral: ±2.0 mm, and ventral: −1.8 mm relative to the bregma). The electrode on each mouse’s head was connected to the headstage and cable (Plexon, Dallas, TX). Local field potentials (LFPs) were recorded by the OmniPlex Neural Data Acquisition System (Plexon, Dallas, TX). Animal behaviors were monitored with a digital camera mounted above (80 cm) the apparatus. Neuroexplorer v3.0 (Plexon, Dallas, TX) was used to assess the power spectra of the LFP signals (filtered 0.1–200 Hz).

#### Immunofluorescence staining and quantification

Mice were anesthetized and transcardially perfused with PBS followed by 4% paraformaldehyde (PFA, Biosharp, BL539A). Brains were removed and fixed in 4% PFA overnight at 4°C, and then consecutively transferred to 20% sucrose and 30% sucrose in PBS for at least 2 days. Samples were embedded in OCT and sectioned into 40 μm coronal slices. Brain sections were preserved in antifreeze solution (40% PBS, 30% glycerol, 30% ethylene glycol) at −20°C till used for immunofluorescence staining.

Brain sections or cultured neurons (fixed with 4% PFA) were permeabilized with 0.3% Triton X-100 diluted in PBS solution for 20 min, washed three times with PBS, and blocked with 5% BSA in PBS for at least 30 min at room temperature. Then, samples were incubated with primary antibodies overnight at 4°C, followed by incubation with secondary antibodies at room temperature for 2 h in the dark, followed by three additional washes with PBS. The brain sections or coverslips were mounted onto slides with antifade mounting medium containing DAPI (Beyotime, P0131). Images were obtained with automatic slice-scanning system (Olympus, SV200) and an orthotopic single-photon confocal microscope (Zeiss Carl LSM 800, Germany).

All brain sections used for analysis were within the bregma −1.7 to bregma −1.94 range. To quantify immunostaining, images were captured using consistent exposure settings across all samples, with three or four sections per mouse analyzed. Cell counts of Flag^+^/GFP^−^ in the hippocampus and cortex were manually determined for each image. The average cell counts of Flag^+^/GFP^−^ in 3 or 4 images of each mouse were analyzed and normalized to 3Flag+Con group. For sections stained with NeuN, the focus was on the hippocampal CA1 region, as structural disorganization in the dentate gyrus (DG) complicated the quantification of overall hippocampal fluorescence intensity. For sections stained with GFAP, Iba1, and AT8, the entire hippocampus was analyzed. Regions of interest (ROI) were defined, and immunostaining intensity was measured using ImageJ. Background signals were subtracted to correct for non-specific staining, and area of positive staining were quantified.

#### Skeleton analysis of microglia

Images of Iba1-stained microglial cells were randomly selected from the bilateral hippocampal CA1 region and analyzed using ImageJ. The images were first converted to 8-bit grayscale, followed by background noise removal and binarization using the Threshold tool. The Analyze Skeleton plugin (https://imagejdocu.tudor.lu/) was then used to measure total process length and the number of branches in the skeletonized images. Cell size was determined using the ImageJ measurement tool. For each group, three or four sections per mouse were analyzed.

#### Open field test

Open field behavior was tested in a white open apparatus (50 cm × 50 cm × 40 cm). The mice were placed in the center of the field and allowed to explore freely for 5 min. Between each experiment, the device was wiped with 75% alcohol to avoid odoriferous cues. The total distance traveled was recorded to evaluate the movement ability of the mice, and the time spent in the central area was measured to detect the anxiety level of the mice.

#### Data processing

Publicly available transcriptomic datasets were retrieved from the Gene Expression Omnibus (GEO). These comprised: (i) hippocampal and cortical samples from healthy and patients with seizure, and including GEO: GSE181804, GSE60862, GSE186334, GSE63808, GSE134697. (ii) the AD cohort GEO: GSE84422 (temporal cortex, with accompanying clinical metadata), and (iii) Raw files and RNA-seq count matrices were downloaded with the “GEOquery” package and processed in R. Platform- or batch-specific variation was mitigated with the “ComBat”.

#### Data analysis

For GEO: GSE181804, GSE60862, GSE186334, GSE63808, and GSE134697, batch-specific variation was mitigated with the “ComBat” and differentially expressed genes (DEGs) were identified using “limma” with thresholds FC ≥ 1.1 and FDR <0.05. Weighted gene co-expression network analysis (WGCNA) was then performed to detect modules most strongly associated with seizure status. Core gene selection was carried out with 7 machine-learning algorithms—ElasticNet, Logistic regression, PLS, Ridge, random forest (RF), neural network (NNET) and generalized linear model (GLM) using “gbm” “randomForestSRC”, “glmnet”, “caret” R package. Least absolute shrinkage and selection operator (LASSO) regression further reduced the panel to 22 core genes. Functional annotation of these genes employed “ClusterProfiler” for GO, KEGG, and gene set enrichment analysis. Correlations between core-gene expression and Braak stage, neurofibrillary-tangle burden and other clinical characters were tested in GEO: GSE84422.

### Quantification and statistical analysis

All data were presented as mean ± SEM and analyzed by GraphPad Prism 8 (GraphPad Software Inc., San Diego, CA, USA). Data were subjected to normality and lognormality tests before comparison. One-way or two-way analysis of variance (ANOVA) followed by Tukey/Sidak’s multiple comparisons test was used in this study. Comparisons between two groups were analyzed by unpaired two-tailed *t* test. For analysis of the correlation, Pearson (data with normal distribution) correlation coefficient was calculated. Survival curves were calculated by implementing a Log rank (Mantel-Cox) test. *p* < 0.05 was considered significant, and ns indicated no significant difference.

### Additional resources

Not applicable. This study has not generated or contributed to a new website/forum and is not part of a clinical trial.
