# Supplementary material for: Tau spread plays an etiopathogenic connection between seizures and Alzheimer’s disease
Source: iScience. 2026 Nov 3;29(11):117747. doi: 10.1016/j.isci.2026.117747 (PMC13755967; doi:10.1016/j.isci.2026.117747)
Supplement: Supplementary file 1 [file mmc1.pdf]

iScience, Volume *29*

Supplemental information

Tau spread plays an etiopathogenic connection between seizures and Alzheimer's disease

Yanli Jiang, Liulin Wu, Wensheng Li, Longfei Li, Zhizhou Huang, Yuran Gui, Jian-Zhi Wang, Rong Liu, Chao-Hui Jing, and Xiaochuan Wang

Supplementary materials

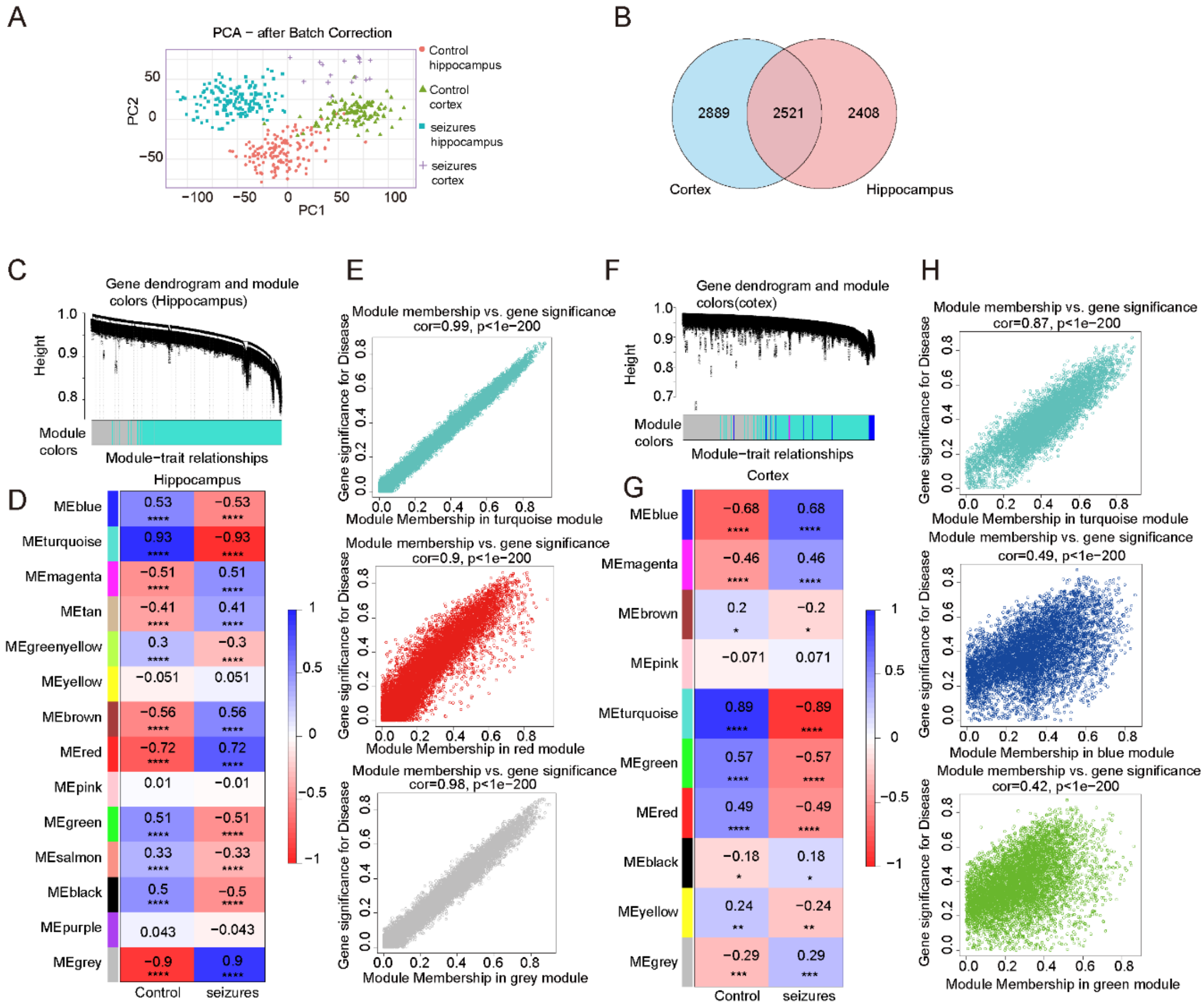

Supplementary Figure 1. Integration of multiple seizures datasets.

(A) Principal component analysis (PCA) after ComBat batch correction. **(B)** Venn diagram showing 2521 overlapping genes consistently differentially expressed in cortex and hippocampus. **(C)** Gene dendrogram and module assignment in the hippocampal dataset by weighted gene co-expression network analysis (WGCNA). The y-axis represents clustering height; the coloured bar displays detected modules. **(D)** Heatmap of correlations between module eigengenes (MEs) and phenotype (Control vs Seizures) in the hippocampus. Numbers and colour intensity indicate Pearson's correlation coefficient, ****p < 0.0001. **(E)** Scatter plots of gene significance (GS) versus module membership (MM) for top 3 hippocampal modules (turquoise, red, and grey). **(F)** Gene dendrogram and module assignment in the cortical dataset. **(G)** Heatmap of ME–phenotype correlations in the cortex. *p < 0.05, **p < 0.01, ***p < 0.001; ****p< 0.0001. **(H)** GS–MM scatter plots for top 3 cortical modules (blue, turquoise, green).

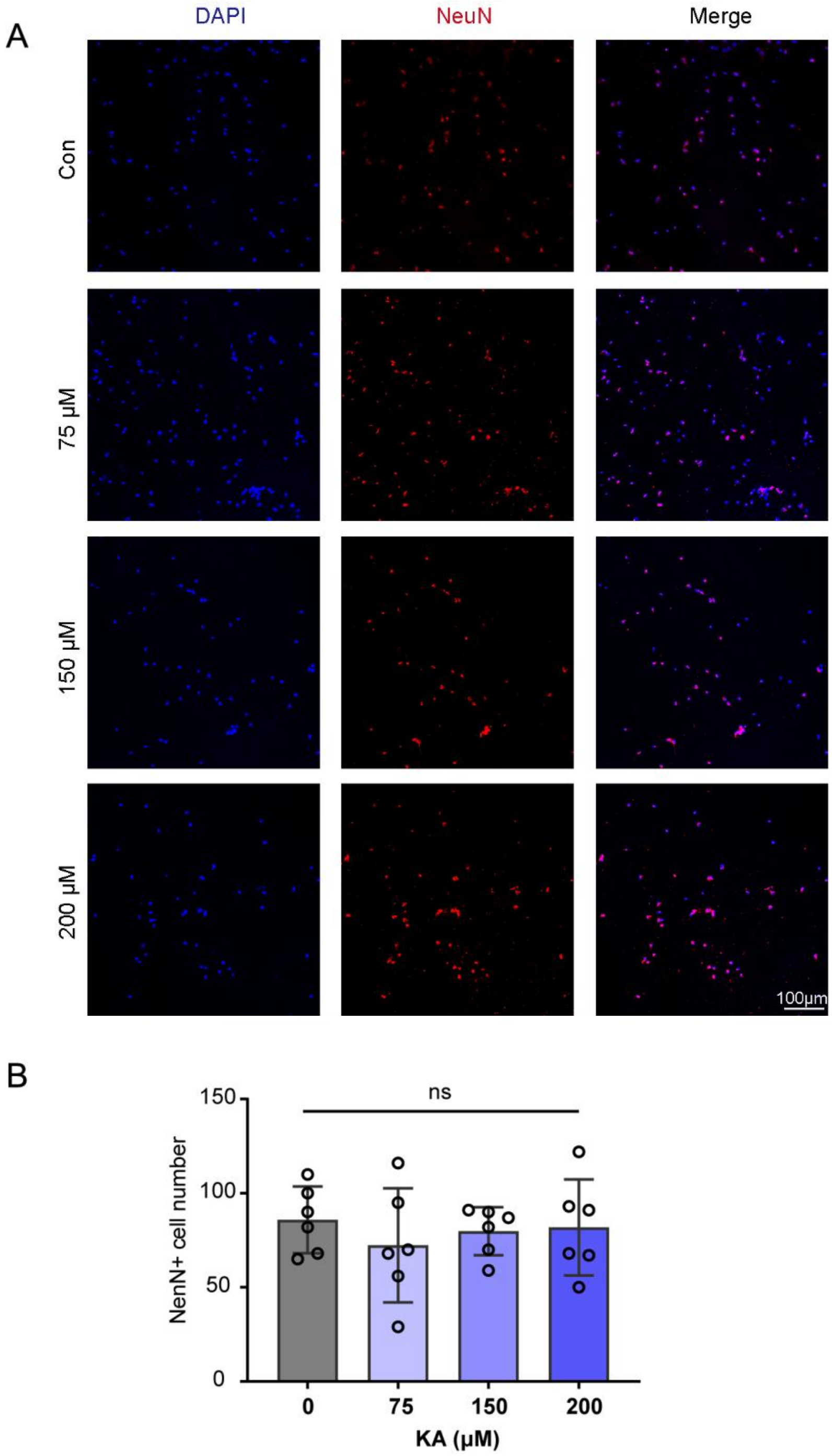

Supplementary Figure 2. KA-induced neurotoxicity analysis in primary mouse neurons. (A) Representative immunofluorescence images stained NeuN after 24 h KA intervention at indicated concentrations. Scale bar = 100 μm (B) Quantification of NeuN-positive neuronal cell numbers among different KA-treated groups. (n = 6 per group). Significant differences were determined using one-way ANOVA followed by Tukey's multiple comparison test. Data are presented as the mean ± SEM, with ns, no significance.

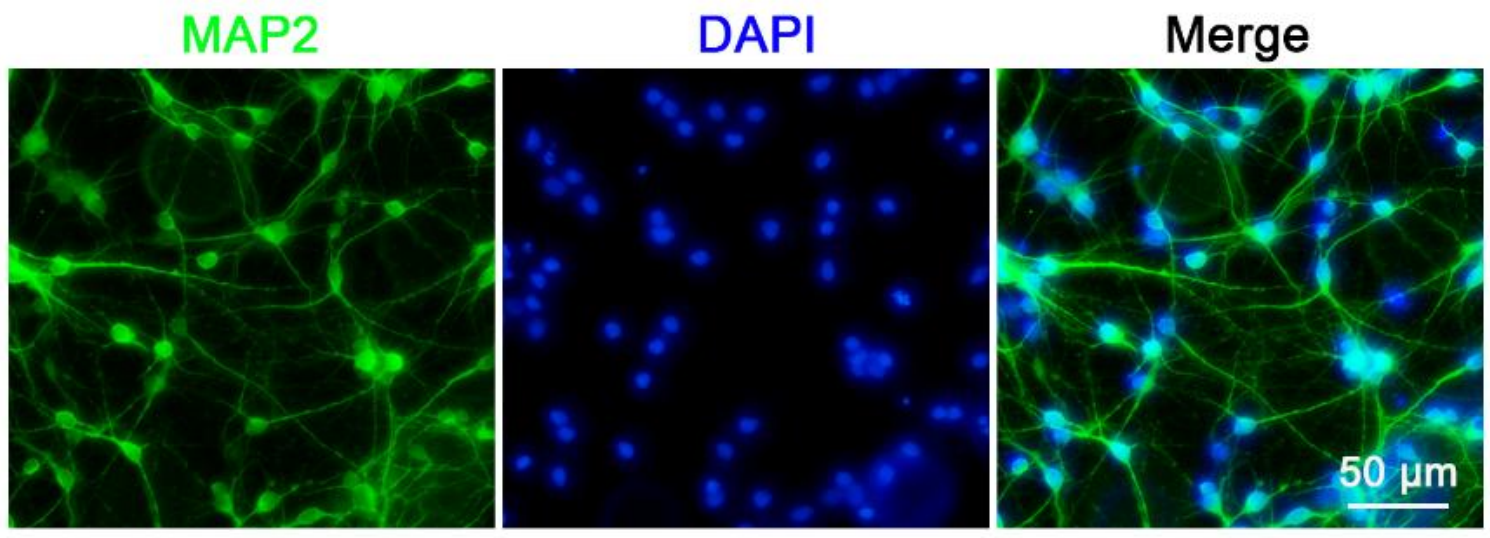

Supplementary Figure 3. Neurons derived from hiPSCs. Immunofluorescent staining of differentiated neurons from hiPSCs using MAP2 antibody. Scale bar = 50 μm.

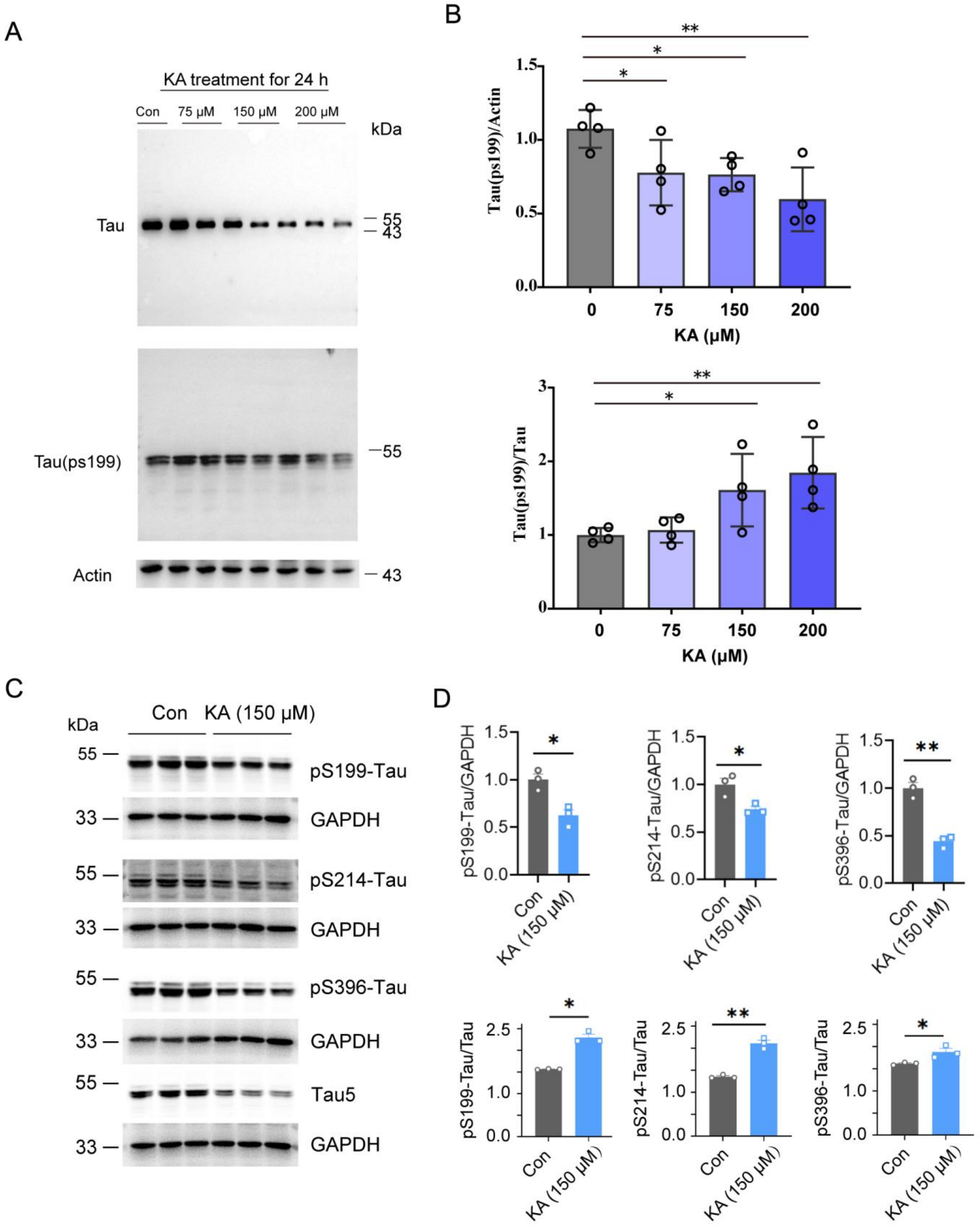

Supplementary Figure 4. KA treatment induces intracellular total Tau and phosphorylated Tau decrease in mouse primary neurons. (A) Western blot detection of total Tau and phosphorylated Tau at Ser199 site in mouse primary neurons treated with gradient concentrations of KA (0, 75, 150, 200 μM) for 24 h. (B) Quantitative analysis of pS199-Tau/Actin and pS199-Tau/Tau among different KA concentration groups. (n = 4 per group). **(C)** Mouse primary neurons were treated with PBS (Con) or 150 μM KA for 24 h, then western blotting was performed with antibodies against pS199-Tau, pS214-Tau, pS396-Tau, Tau5 and GAPDH. **(D)** Relative pS199-Tau/GAPDH, pS214-Tau/GAPDH, pS396-Tau/GAPDH, Tau5/GAPDH， pS199-Tau/Tau, pS214-Tau/Tau, and pS396-Tau/Tau. (n = 3 per

group). Significant differences were determined using one-way ANOVA followed by Tukey's multiple comparison test for multiple-group comparisons in Panel B. For two-group comparisons in Panel D, unpaired two-tailed t-test was applied when variances were homogeneous, whereas Welch's unpaired two-tailed t-test was used for data with unequal variances. Data are presented as the mean ± SEM, with *$p < 0.05$, and **$p < 0.01$, ns, no significance.

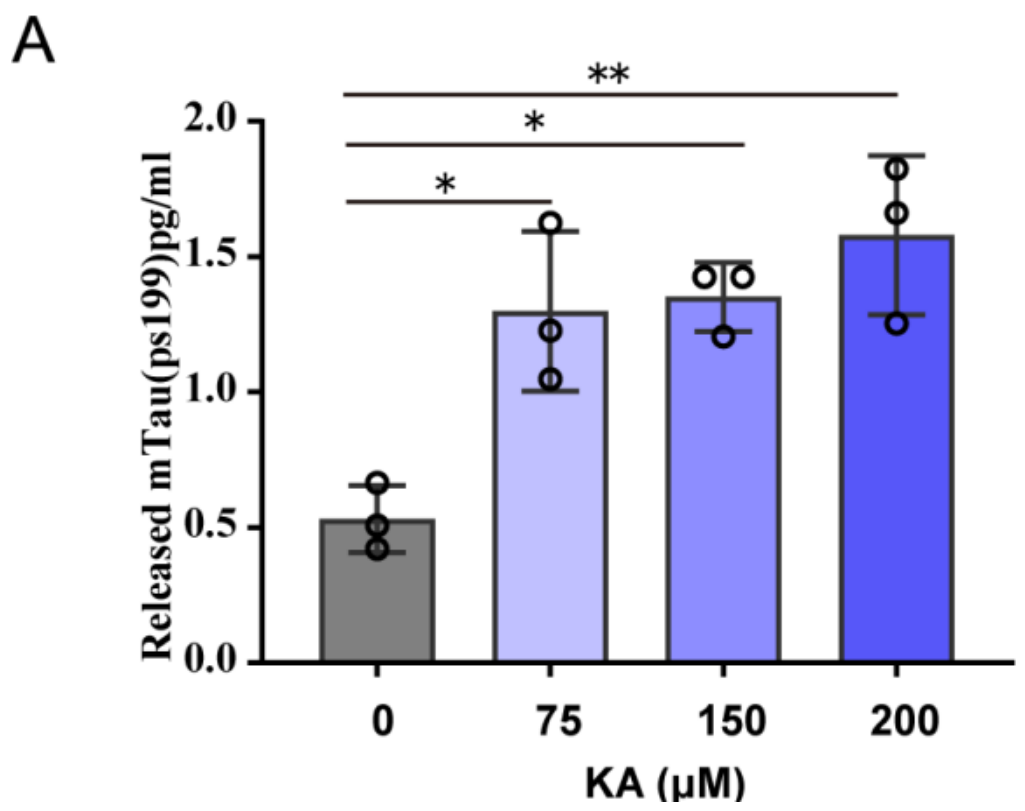

Supplementary Figure 5. Dose-dependent secretion of phosphorylated Tau induced by KA in primary mouse neurons. (A) ELISA detection of extracellular released pS199-mTau levels (pg/mL) in culture supernatant after 24 h treatment with gradient KA concentrations. Statistical analysis: one-way ANOVA with Tukey's post-hoc test, *p < 0.05, **p < 0.01.

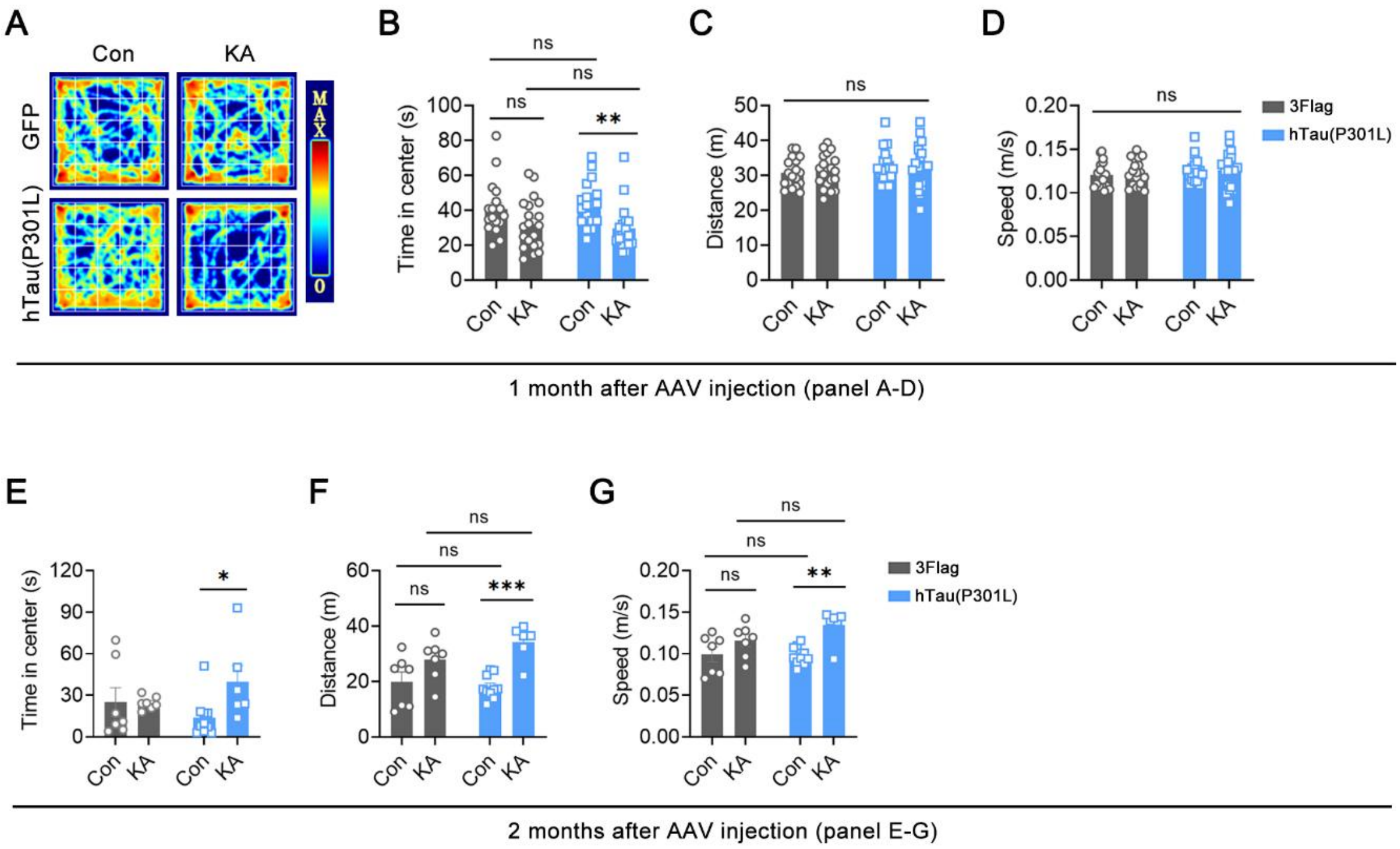

Supplementary Figure 6. Motor activity is increased in mice with Tau pathology and MTLE comorbidities. (A-D) Open field test was performed after virus injection for 1 month (GFP + Con, *n* = 18; GFP + KA, *n* = 20; hTau (P301L) + Con, *n* = 18; hTau (P301L) + KA, *n* = 19). **(A)** Trajectories of the mice in the open field test. **(B)** Time spent in the central region, **(C)** distance traveled, and **(D)** speed were quantified in the open field test. **(E-G)** Open field test was performed after virus injection for 2 months (GFP + Con, *n* = 7; GFP + KA, *n* = 7; hTau (P301L) + Con, *n* = 10; hTau (P301L) + KA,

n = 5-6). **(E)** Time spent in the central region, **(F)** distance traveled, and **(G)** speed were quantified in the open field test. Significant differences were determined using two-way ANOVA followed by Sidak's multiple comparison test. Data are presented as the mean ± SEM, with *$p < 0.05$, **$p < 0.01$, ***$p < 0.001$, ns, no significance.

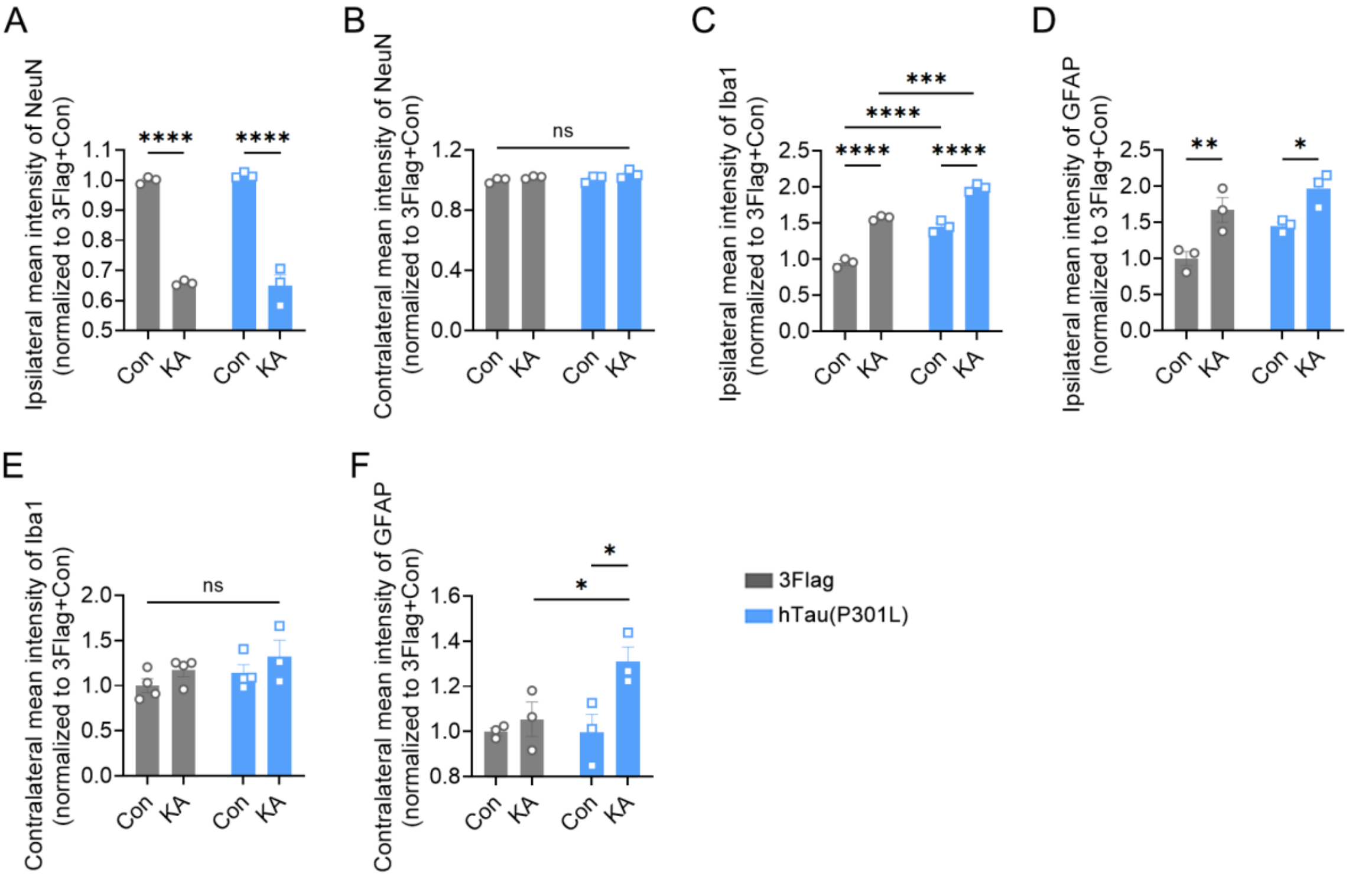

Supplementary Figure 7. Seizures induce neuronal loss and gliosis in the contralateral hippocampus of hTau (P301L)-overexpressing mice. (A, B) Mean intensity of NeuN in the ipsilateral and contralateral hippocampus were quantified and normalized to 3Flag + Con group. **(C, D)** Mean intensity of Iba1 and GFAP in the ipsilateral hippocampus were quantified and normalized to 3Flag + Con group. **(E, F)** Mean intensity of Iba1 and GFAP in the contralateral hippocampus were quantified and normalized to 3Flag + Con group. Significant differences were determined using two-way ANOVA followed by Sidak's multiple comparison test (*n* = 3 mice per group; 3-4 brain slices per mouse). Data are presented as the mean ± SEM, with *$p < 0.05$, **$p < 0.01$, ***$p < 0.001$, and ****$p < 0.0001$, ns, no significance.

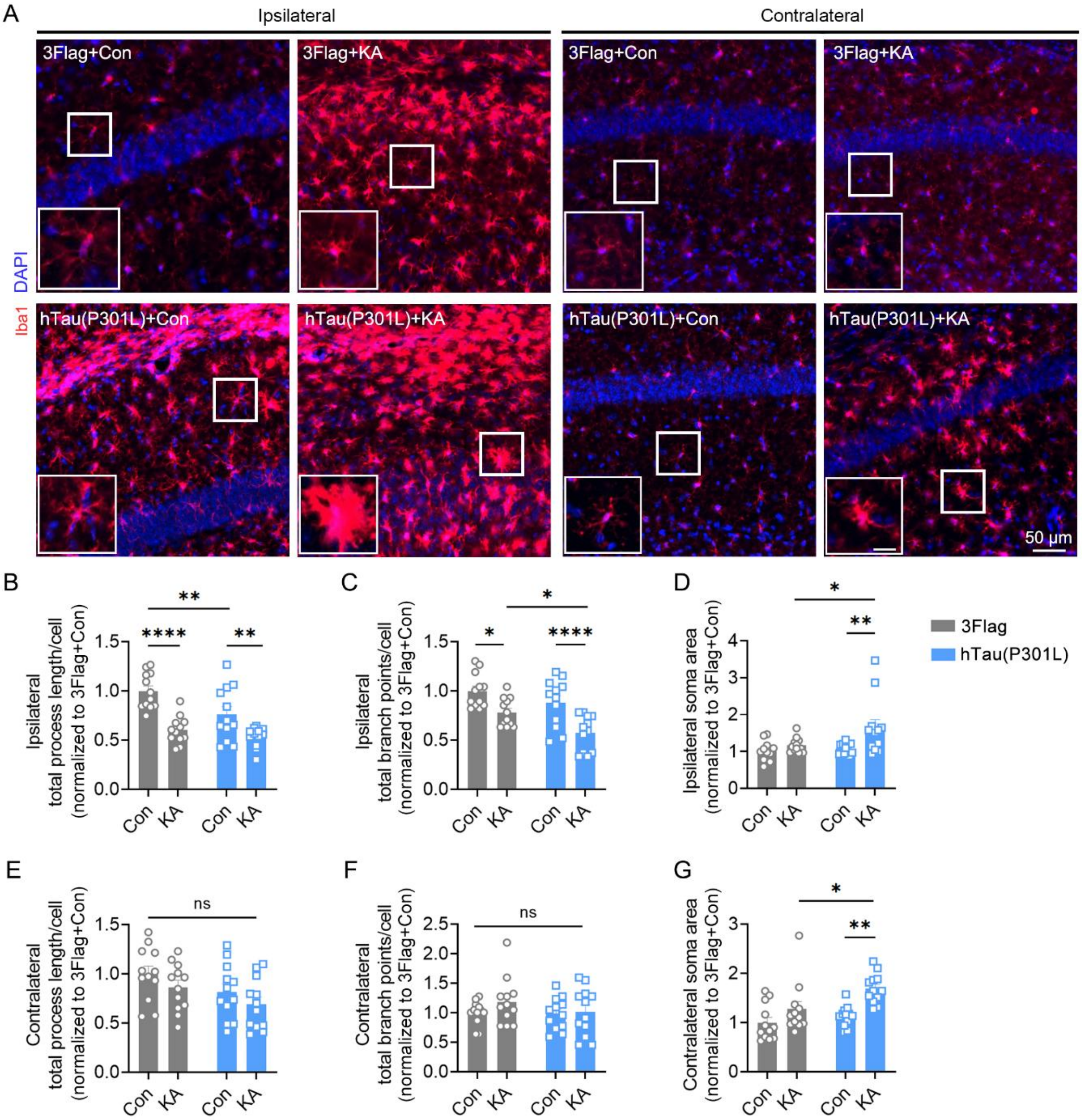

Supplementary Figure 8. Seizures induce contralateral hippocampal microglia activation in hTau (P301L)-overexpressing mice. (A) Representative immunostaining images of Iba1 positive microglia (red) in different groups of bilateral hippocampal CA1 region. Scale bar = 50 μm and zoom in images bar = 20 μm. **(B-G)** The histogram represents the quantification of process length (B, E), number of total branch point per cell (C, F) and soma area (D, G) of Iba-1 cells in the bilateral hippocampal CA1 regions of different groups. Significant differences were determined using two-way ANOVA followed by Sidak's multiple comparison test. (n = 4 mice per group; 3 sections per mouse). Data are presented as the mean ± SEM, with *$p < 0.05$, **$p < 0.01$, and ****$p < 0.0001$, ns, no significance.
